# Inequality of Opportunity in Tertiary Education: Evidence from Europe

**DOI:** 10.1007/s11162-021-09658-4

**Published:** 2021-10-20

**Authors:** Flaviana Palmisano, Federico Biagi, Vito Peragine

**Affiliations:** 1 Department of Economics and Law, Sapienza University of Rome, Via del Castro Laurenziano 9, 00161 Rome, Italy; 2 JRC European Commission, Ispra, Italy; 3 University of Bari, Bari, Italy

**Keywords:** Inequality of opportunity, Higher education, Europe, Social inequality, D63, I2, C14

## Abstract

This study provides comparable lower-bound estimates of inequality of opportunity for tertiary education (EIOp) for 31 countries in Europe, by using the two EU-SILC waves for which information on family background is available (2005 and 2011). The results reveal an important degree of heterogeneity, with Northern European countries showing low levels of inequality of opportunity and Mediterranean and Eastern European countries characterized by significant degrees of unfair educational inequalities. Parental education and occupation are the most relevant circumstances in the great majority of the countries considered. This study also exploits the two point-in-time observations available and analyses the relationship between some country-specific characteristics and inequality of opportunity in tertiary education. The analysis documents a negative association between EIOp and real GDP per capita, possibly indicating that higher equality of opportunity in tertiary education and economic growth are complementary objectives. Two results emerge as especially robust: in all the specifications we find a positive association between EIOp and the students/teacher ratio, and a negative one between EIOp and public spending in tertiary education. While we do not claim that such correlations should be interpreted causally, we think that they might indicate a meaningful underlying relationship between equality of opportunity in tertiary education and the availability of financial and non-financial resources.

## Introduction

Educational attainment and, more generally, human capital, are among the main determinants of social progress. In the past, compulsory primary and lower secondary education have guaranteed to children from less privileged families the possibilities of moving up the social ladder. As economies in the industrialized world have become more knowledge-based, research/innovation activities and human capital have become central. In fact, human capital—and hence education—has been identified as one of the engines of growth, for countries that are closer to the technological frontier (which compete by pushing further the frontier) and for those that are still distant from it (for which improvement in absorptive capacity might be more relevant). Irrespective of the level of technological progress of a country, upper secondary and tertiary education are recognized among the most relevant institutions determining whether individuals and countries make or break the challenges posed by the globalized modern knowledge economy. However, upper secondary and tertiary education cannot be seen just with the lenses of economic efficiency. Both have a very important egalitarian role, as they contribute to render societies more mobile. Increased access to upper secondary education and to tertiary education improves the opportunities of individuals from under-privileged background; this can produce positive effects on efficiency as well, since access to the highest positions and responsibilities in society depends also on merit and not only on socio-economic status. In fact, as long as education expansion does not happen at the expenses of its quality, there is really no trade-off between efficiency and equity.

In such context, our study focuses on tertiary education, as we believe that increasing the opportunities for higher education creates the basis for the foundation of a more mobile and equitable society, reducing the influence that parental background exerts on students’ future socio-economic outcomes. In practice, tertiary education is seen as a fundamental instrument to break the transmission of disadvantage from one generation to the next. Due to the strong relationship between education and earnings, equality of opportunity in education outcomes can also increase intergenerational mobility in earnings.^1^

We investigate the extent of inequality of opportunity in tertiary education (EIOp hereafter) in Europe using data from EU-SILC 2005 and 2011.

The concept of equality of opportunity adopted here is the one flourished in the field of normative economics and distributional analysis in the last two decades.^2^ This literature has developed concepts of fairness for the context in which individual achievements are partly the outcome of circumstances outside individual control (such as ethnicity, gender, location of birth, family and social background) and partly the consequence of individual efforts, which are expressions of personal responsibility.

Those concepts revolve around the idea that inequalities due to circumstances are unfair and should be eliminated as much as possible, while inequalities that result from unequal efforts are acceptable. This literature has witnessed a rapidly growing number of empirical applications interested in measuring the degree of inequality of opportunity (IOp) and in evaluating public policies in terms of equality of opportunity, mainly in the context of income distributions (see, among others, Aaberge et al., 2011; Checchi & Peragine, 2010; Lefranc et al., 2009; Roemer et al., 2003).

In the field of education, the principle of equality of educational opportunities is often referred to as the leading normative principle for all those who consider educational achievements to be relevant in their own right (see Ferreira & Gignoux, 2014). EIOp matters also from a positive perspective: the distribution of educational achievements, in fact, plays a role in the distribution of earnings (Blau and Khan, 2005), as predicted by the human capital theory, and influences economic growth (Hanushek & Wößmann, 2008, 2010).

Because of its normative and positive relevance, an increasing number of contributions investigate equality of opportunity in education. Most of these contributions focus on the relationship between pupils’ circumstances and inequalities in standardized test scores measured in international surveys, such as TIMSS (Trends in International Mathematics and Science Study), PIRLS (Progress in International Reading Literacy Study), and PISA (Programme for International Student Assessment), regularly conducted across different groups of countries (see, among others, Betts & Roemer, 2005; de Carvalho et al., 2012; Lasso de la Vega et al., 2019; Ferreira & Gignoux, 2010, 2011, 2014; Gamboa & Waltenberg, 2012; Hashemi & Intini, 2015; Peragine et al., 2015; Salehi-Isfahani et al., 2014; Schütz et al., 2008). Using such data sources allows for consistent cross-country comparisons as they provide standardized measures of achievements and the same set of information at individual and school levels.

Inequality of educational opportunity for higher education is less investigated in the context of the equality of opportunity literature, partly because of the scarcity of adequate information. Standardized tests are largely non-existent, even at national level, and the measurement of relevant circumstances is more difficult than in primary and secondary education, because often students in higher education have left the family in which they grew up. Exceptions are Peragine and Serlenga (2008) and Brunori et al. (2012), who propose an analysis of inequality of opportunity for tertiary education in Italy. Using data on final graduation marks and on the earnings of individuals with a tertiary education degree, Peragine and Serlenga (2008) find strong family effect on the performances of students enrolled in university as well as on graduates’ transition to the labour market. Brunori et al. (2012) analyze equality of opportunity in access to tertiary education and find evidence of a reduction in EIOp, especially between 1998 and 2001. More recently, Jaoul-Grammare and Magdalou (2013) analyze the French higher education system by comparing the situations in 1992 and 2004. They find evidence of inequality of opportunity for tertiary education in each of the two years, with an increasing trend during the reporting period.

In this study, building on the theory of equality of opportunity referred above, we adopt the conceptual framework developed for the definition of inequality of opportunity in income and we adapt it so as to formalize the concept of inequality of opportunity in tertiary education, hence obtaining a consistent measurement strategy.

We choose completion of tertiary education as an outcome variable à la Roemer (1998) because education is a crucial generator of well-being, both directly and indirectly.

In general, educational achievement is an important determinant of future earning capacity and, thereby, of individuals’ welfare (Psacharopoulos & Patrinos, 2018). In particular, tertiary education completion increases the probability of accessing highly remunerated jobs. In fact, at least in developed countries, as a consequence of the increase in mandatory years of schooling,^3^ higher education is gaining growing attention among scholars and policy makers: Psacharopoulos and Patrinos (2018) estimate private returns to higher education of around 12% in high-income countries in the 2000s.^4^

Educational attainment also affects well-being through its impact on a variety of non-income variables such as health, fertility, crime, political attitudes and social behavior in general (Oreopoulos & Salvanes, 2011).

Last, by choosing tertiary education completion as the outcome variable, it is possible to overcome the challenge of distinguishing between circumstances and effort, which in the case of primary and (lower) secondary school is more problematic. In fact, because of their young age, students attending primary or (lower) secondary schools can hardly be held responsible whatever their actions are in relationship to education, so that all determinants of educational attainment should be considered circumstances.^5^ On the contrary, in the case of tertiary education, which students enter as adults, it is possible to distinguish clearly between circumstances and factors under personal control.

We are conscious that by concentrating on completion of tertiary education we overlook other aspects—such as enrollment, type of degree, prestige of institutions, field of study, and the geographic location of institutions—that might have an impact on individual’s future earnings prospects. However, our choice, which is in tune with the economic role recognized to education in mainstream economic theory—i.e. as an investment in human capital (Becker, 1993)—is appropriate for the type of research question addressed here. Our interest is not in estimating returns to tertiary education, conditional on individuals characteristics (in that case controlling for, say, field of study and university ranking would be highly advisable). Our aim here is more modest, as we only focus on the (conditional) probability of completing higher education, which we take as a general proxy for higher well-being (relative to lower levels of education). We aim at measuring inequality in the distribution of such outcome variable, distinguishing between fair and unfair inequality (i.e. inequality of opportunity).

The approach to (in)equality of opportunity adopted in this study is related to, but also someway different from, the one typically found in sociology of education^6^ or education studies. Following the seminal work of Boudon (1974), many sociologists have devoted attention to the role played by socio-economic background on educational choices (e.g. in relationship to upper secondary education—vocational vs general—and tertiary education/field of study), educational outcomes (highest educational attainment level vs results on standardised tests^7^) and labour market outcomes (expressed in terms of occupation, income, social class, etc.; see Hadjar, 2019; Dollman, 2019; Schneider, 2018; Breen & Jonsson, 2005).

Following Bourdieu’s (Bourdieu, 1986) notion of cultural capital, Farkas^8^ develops a theory of social reproduction based on the interaction between parents and teachers, where the latter, through their evaluation of students’ performance and attitudes, influence students’ academic future. Middle and high social class parents are better equipped to understand the metrics adopted by teachers in their students’ evaluation^9^ and hence more successful in endowing their offsprings of those “cultural capital” elements that are useful to succeed in education and in life. In addition, more educated parents are typically more able to support their children’ out-of-school learning (including with private tutoring), and to positively affect their educational aspirations.

Alternative explanations for the socio-economic (and racial) gradient in educational attainment point to the importance of the opportunity cost of education (including its financing), which, in the presence of credit constraints (and of higher chances of employment in highly paid occupations^10^ for privileged students), can explain why individuals with a disadvantaged background might “rationally” invest less in human capital. Other “rational” theories posit that students (and families) make their educational choices based on relative risk aversion (see Breen & Goldthorpe, 1997): they intend to minimize the risk of downward social mobility (often defined in terms of parental occupation). Since such risk tends to increase with the social position one starts from, individuals from a disadvantaged social background (i.e. with low parental occupational attainment) tend to “invest” less in their own education because, even with a low “investment”, they are unlikely to fall down on the social ladder (while those starting from the top have everything to lose).

A partially complementary attempt to account for social stratification in (higher) education posits that affluent parents put in place strategies meant to assure to their offsprings a competitive advantage in the race for life-success by adjusting both the quality and the quantity of their educational investment (Lucas, 2001; Lucas & Irwin, 2018). In times of mass education this implies that rich parents will try (and often succeed) to direct their children towards the more prestigious and remunerative tracks and schools. With the consequence that measuring education with a simple indicator such as educational attainment is not sufficient. To fully understand how social stratification in education is built, one would need to take into account qualitative elements (e.g. the quality of the school/university attended, its vocational vs academic nature, whether it is private or public, etc.) and this should be done along the entire educational career^11^,^12^ (long-lasting consequences are often the result of choices taken at an early age).

(In)equality of opportunity in higher education is the result of many different factors, ranging from educational policies and reforms to productivity growth and technological progress, the functioning of the welfare state, demographic factors and political conflict. Identifying the impact of each driver is extremely complex and data intensive, and it also requires in-depth knowledge of the evolution of education policies in a given country. This also explains why studies that adopt a comparative approach tend to focus on time trends of correlation coefficients between socio-economic status and educational attainment (or achievement). In the aftermath of World War II, most European (and industrialized) countries were characterized by reforms aiming at universal education, which increased the age of mandatory education, and led to investments in secondary education expansion and to reductions in its private costs (at least for the lower-secondary level). The evidence (Breen et al., 2009, 2010) shows that, Europe—in the period 1950–1975—has witnessed a decreasing association between social origin^13^ and educational attainment (i.e. by lower levels of inequality of educational opportunities^14^). This phenomenon has been especially driven by the transition from primary to secondary education. A recent study (Barone & Ruggera, 2018), extending the analysis of Breen and co-authors by increasing the number of countries and using more recent data, confirms the reduction in inequality of educational opportunities in 26 European countries for the cohorts born in 1930–1944 and 1945–1954. At the same time, the authors find that, for more recent cohorts (1955–1964 and 1965–1980), the process of reduced association between socio-economic status and parental background has weakened and, in some countries, even stalled. As for tertiary education, Barone and Ruggera (2018) find that in some countries the role of parental background has decreased while in others it has remained fairly stable.

The main element that these studies have in common is that, given the selection of the outcome variable (which partly depends on data availability), the empirical analysis tests whether, conditional on a set of observable characteristics, socio-economic background (i.e. parental education/occupation/social class/income) has an impact on the outcome variable. In practical terms, when the coefficient on the proxy for socio-economic background is statistically significantly different from zero, the conclusion is that inequality of opportunity exists, because descendants’ outcomes are affected by their socio-economic background. In some cases the analysis goes further and researchers try to identify how certain characteristics of the education system affect inequality in the relevant educational outcomes. Such an approach is often used in comparative studies, in which across-country/regions institutional differences are used as an approximation to quasi natural experiments.^15^ A typical example of an institutional feature leading to socio-economic-driven educational inequality is early tracking (see Skopek et.al, 2019; Van de Werfhorst, 2019; Jerrim et al., 2019; Meschi & Scervini, 2014; Brunello & Checchi, 2007).

Our approach shares with the one just described the attention paid to factors conditioning the individual’s educational performance, which are mostly related to her/his socio-economic background (but not exclusively). However it also differs from the latter, for two main reasons. First, the focus is not on socio-economic background alone, but on factors that are outside individuals’ control (among which socio-economic background certainly plays a major role). Second, since our aim is to distinguish between the ethically acceptable and the un-acceptable parts of observed inequality, we use an indicator to pinpoint how much of the observed outcome inequality can be accounted for by factors that are outside individual’s control, interpreting this as a lower-bound measure of ethically unacceptable inequality in tertiary education attainment (hence our approach combines normative and positive aspects).

Results show that there are large across-country differences, with Nordic countries performing much better than Mediterranean and Eastern countries. In order to shed some light on the drivers and consequences of such differences, we provide a decomposition of our estimates of EIOp, which shows that, in almost all the countries analyzed, parental education and parental occupation represent the most important circumstances. Next, we regress measured EIOp^16^ on a set of indicators that capture different characteristics of the countries’ educational systems, labour markets, and economic performance. We find evidence that real GDP per capita is negatively associated with EIOp, indicating that equality of opportunities and growth are not contrasting objectives. We also find that a higher students/teacher ratio is associated with higher EIOp (see Brunello & Checchi, 2007) and that a robust negative correlation exists between public expenditure for tertiary education and EIOp. Lastly, we argue that interventions aimed at reducing EIOp are desirable, as this kind of inequality has an impact on life-cycle achievements. Indeed, our data show that EIOp is strongly and positively associated to IOp in income.

To the best of our knowledge, this is the first paper (i) to provide cross-country lower-bound estimates of IOp for tertiary education in Europe and (ii) to shed some evidence on the relationship between EIOp and characteristics of the educational systems and institutions. Concerning (ii), because of data limitation, a causal inference analysis is out of scope. We rather put the focus on some of the different institutional features that are associated to countries with different levels of educational inequality of opportunity.

The study is structured as follows. Section Assessing Inequality of Opportunity in Tertiary Educationoutlines the methodology used to analyze inequality of opportunity in tertiary education. Section Data describes the data. Section Measuring Inequality Of Opportunity In Tertiary Education presents the results of EIOp estimates. Section Exploring Drivers of EIOp presents the results of the correlation analysis and the relationship between EIOp and IOp in income. Section Discussion concludes.

## Assessing Inequality of Opportunity in Tertiary Education

The EOp “canonical model” (see Ferreira & Peragine, 2016) suggests to interpret an individual’s outcome ($x$) as the result of two different set of variables: circumstances ($C$)—which are outside individual control and for which she cannot be held responsible—and effort ($e$)—which, on the contrary, is under individual control, and for which she is fully accountable. This can be written, for each individual, as:

1 $$x=g(C,e)$$

Equation (1) can be seen as a reduced-form model in which outcomes are exclusively determined by circumstances and effort. In the context of our analysis, the outcome variable $x$ refers to the completion of tertiary education. Equality of opportunity requires differences in outcomes due to differences in circumstances to be eliminated, while differences due to effort are deemed to be ethically acceptable. Given that we cannot directly characterize the opportunity sets available to individuals, we focus on measuring inequality of opportunity, with a two-step procedure consistent with an ex-ante approach.^17^ Its reduced data requirements make this approach widely used in empirical analyses. First, the actual distribution of the outcome variable $\left[ {{\mathbf{X}}_{ij} } \right]$ is transformed into a counterfactual distribution $\left[ {{\tilde{\mathbf{X}}}_{ij} } \right]$ that reflects only and fully the unfair inequality in $\left[ {{\mathbf{X}}_{ij} } \right]$, while all the fair inequality is removed. In the second step, a measure of inequality is applied to $\left[ {{\tilde{\mathbf{X}}}_{ij} } \right]$. Following Peragine (2002), Checchi and Peragine (2010) and Ferreira and Gignoux (2011), the counterfactual distribution $\left[ {{\tilde{\mathbf{X}}}_{BT} } \right]$ is obtained parametrically by estimating the individual outcomes $x_{ij}$ as function of circumstances only. Ultimately, this captures the extent to which circumstances—both directly and indirectly—contribute to the variation observed in the outcome variable (in our case completion of tertiary education). For example, parents’ level of education may influence both directly and indirectly an individual educational attainment, through additional resources available for higher education (a direct effect) and through cultural and role models (the indirect effect).

In practice, a predicted outcome based purely on circumstances is constructed for each individual, obtained from the reduced-form regression of tertiary educational attainment on circumstances:

2 $$x=c\beta +\varepsilon$$

Predicted values for completion of tertiary education are used as a parametric approximation to the smoothed distribution ${\widehat{{\varvec{x}}}}_{BT}$ where ${\widehat{x}}_{BT}=c\widehat{\beta }$. Given that the outcome variable is binary (completed tertiary education or not), a probit model is employed to estimate Eq. (2). This approach follows Ferreira and Gignoux (2011), which in turn draws on Bourguignon et al. (2007).

Once the smoothed distribution $\left[ {{\tilde{\mathbf{X}}}_{BT} } \right]$ is obtained, any inequality measure $I$ applied to such a distribution, $I\left( {{\tilde{\mathbf{X}}}_{BT} } \right)$, is to be interpreted as a measure of inequality of opportunity.

A particular measure is required if the outcome of interest is binary – as in our case. A commonly used measure is a “dissimilarity index” (D-index) – broadly speaking, the average distance between predicted outcomes and the mean predicted outcome. Formally:

3 $$D=\frac{1}{N\overline{\widehat{x}}}{\sum }_{i=1}^{N}\left|{\widehat{x}}_{i}-\overline{\widehat{x} }\right|$$

The interpretation of this index is very similar to the Gini index: a dissimilarity index equal to 0 means that opportunities are equally distributed across individuals, a dissimilarity index approaching 1 means that all opportunities are concentrated on one individual. Thus, a low (high) value of $D$ indicates a low(high) level of EIOp. Higher than average predicted outcomes, based on favorable circumstances, will lead to a higher D-index, as will lower than average predicted outcomes due to unfavorable circumstances.

Finally, each circumstance may play a different role in the determination of EIOp. If one or more relevant circumstances are not observable, and if we cannot exclude that they are correlated with observable circumstances, an exact causal identification of the relative role of each circumstance is not possible. However, a description of the relative role of observable circumstances may be of some interest (Ferreira & Gignoux, 2011). In order to describe the specific contribution of each circumstance to EIOp, we make use of a sequence of counterfactual distributions comparisons.

The importance of a given circumstance *i* in generating EIOp is measured by comparing the estimate of EIOp when all circumstances, including *i,* are allowed to vary to the estimate of EIOp when the specific circumstance *i* is held fixed (i.e. we measure inequality with a distribution that eliminates variation across individuals that could arise from that circumstance). The contribution of a given circumstance to inequality may depend on the order in which each circumstance is eliminated. We account for this by implementing a Shapley-value decomposition (see Shorrocks, 2013), assessing all possible elimination sequences and taking the average across the estimated contributions.^18^

We apply this model to the data (described in Section Data) and provide an in-depth assessment of educational inequality of opportunity in the European context (in Section Measuring Inequality of Opportunity in Tertiary Education). Moreover, we use the estimates of EIOp to investigate the channels (i.e. country-specific characteristics) through which circumstances may affect the likelihood of completing higher education. While there exists an extensive literature studying the determinants of income inequality (see among others Perugini & Martino, 2008), the literature on the determinants of inequality of opportunity is scant. To the best of our knowledge, Marrero and Rodriguez (2012) and Checchi et al. (2016) are the only published studies that shed some light on the role of (some) institutional variables on aggregate inequality of opportunity in income in Europe. We mimic their approach and analyse the potential association between certain country-specific characteristics and the EIOp estimates (see Section Exploring Drivers of EIOp).

We consider a variety of indicators that reflect a country’s level of economic (real GDP per capita) and human capital development (the gross enrolment rate in tertiary education; the share of the population older than 25 with at least a secondary level of education; the share of individuals in the age group 15–25 enrolled in vocational education; the share of all secondary education student enrolled in general programmes). We expect that policies supporting secondary educational attainment also favour higher independence between circumstances and tertiary education attainment (i.e. lower EIOp). On the other hand, higher shares of students enrolled in vocational education (and, symmetrically, lower shares of students enrolled in general programmes) are often found in countries characterized by dual systems of education and early tracking, both of which tend to exacerbate the impact of family socio-economic background on educational inequalities (Anders & Henderson, 2019). As for the gross enrolment rate in tertiary education, previous studies (Triventi, 2013) have found that it tends to be positively associated with measures of inequality in higher education, possibly as a result of increased competition for the “tickets” (i.e. university degrees) to future “good” (or relatively “less bad”) jobs.

We also consider a set of variables related to public spending, such as public expenditure on education (as a percentage of total government expenditure and as a percentage of GDP) and public expenditure on tertiary education (as a percentage of total government expenditure and as a percentage of GDP). Quality of education may also play a role, by compensating the disadvantage of students coming from a low socio-economic background. Unfortunately, data on school/university quality are not easily available. More modestly, we have considered the students/teacher ratio in tertiary education and the outbound and inbound mobility ratios (measuring the internationalization of universities) as proxies for quality of education. The students/teacher ratio is often used as a proxy for quality of education, as it signals the amount of teaching resources available to each student (see McDonald, 2013; OECD, 2019). Outbound mobility is used as one proxy for poor quality of higher education, while the opposite holds for inward mobility (in both cases students react by voting “with their feet”), consistent with the results of Van Bouwel and Veugelers (2013).

Finally, we add a group of institutional variables that try to capture the benefits from completion of tertiary education, such as: the ratio between the employment rate of individuals with tertiary education and the employment rate of individuals with secondary education; the ratio between the unemployment rate of individuals with tertiary education and the unemployment rate of individuals with secondary education; the earnings of workers with tertiary education relative to the earnings of individuals with secondary education.

## Data

We use data from the 2005 and 2011 waves of the European Survey on Income and Living Conditions (EU-SILC), which is annually run by national Central Statistics Offices and collects information on the income and living conditions of households in the EU (plus Norway, Iceland and Switzerland). The survey contains information on a large number of individual and household characteristics as well as specific information on poverty and social exclusion. We use the 2005 and 2011 waves because they are the only two waves containing the special module on intergenerational mobility, which includes information on individuals’ circumstances.

Respondents between the ages of 25–60 were asked to provide additional information about their parents’ social and economic situation during their teenage years (in particular, when aged 14–16). These additional modules report information on educational attainment, occupation, as well as the labour market activity status of respondent's mother and father and the presence of financial problems in the household. The 2005 survey includes 26 countries, while the 2011 survey consists of 31 countries.^19^ We restrict our sample to native individuals in order to avoid possible bias in our estimates, which could arise if migrants enter in the destination country when adults (possibly after graduating in the birth country), since the information on their age when migrating is not available in the data.^20^ The main advantage of EU-SILC is the relatively large number of countries covered, which allows for cross-country comparative analysis of inequalities. Some descriptive information concerning the countries included in this analysis is reported in Table 1, where it is possible to observe their high heterogeneity in terms of both mean income and inequality as measured by the Gini index.

**Table 1:** Descriptive statistics

Country	Mean Income (2011)	SD (2011)	Income Inequality (2011)	Sample Size (2011)	Mean Income (2005)	SD (2005)	Income Inequality (2005)	Sample Size (2005)
AT	26,890	14,219	25.70	5549	21,606	11,445	25.40	5678
BE	24,731	24,340	23.71	5148	20,323	11,117	23.67	4915
BG	3807	3059	33.66	7387	n.a	n.a	n.a	n.a
CH	43,428	29,121	27.25	5519	n.a	n.a	n.a	n.a
CY	22,195	16,489	27.28	4099	16,551	10,579	26.26	4552
CZ	9057	5014	25.68	7001	5179	3151	26.59	4805
DE	22,734	15,107	28.12	11,950	20,952	14,392	27.24	13,830
DK	30,462	18,681	26.71	2701	25,939	12,471	21.99	3592
EE	7297	4657	32.84	5048	4084	2962	34.83	4373
ES	18,307	11,845	31.79	14,617	13,503	8337	31.04	16,130
FI	26,777	16,885	25.00	4860	21,491	23,420	25.94	6630
FR	25,204	20,643	28.95	10,224	19,553	10,720	25.99	10,079
GR	14,066	10,237	33.04	5940	12,088	8071	32.14	6549
HR	6719	3957	30.83	6128	n.a	n.a	n.a	n.a
HU	5383	2961	27.74	14,159	4062	2777	28.57	8208
IE	25,575	14,866	29.97	2629	25,225	24,724	32.64	3995
IS	21,430	10,901	23.02	1613	27,643	18,805	24.33	1676
IT	19,521	14,563	31.63	20,364	18,251	14,145	32.17	26,138
LT	4815	3246	34.50	5153	2763	2097	37.43	5118
LU	41,182	20,657	23.44	3495	35,647	17,092	23.22	2405
LV	5475	3861	35.89	6086	2898	2437	36.81	3497
MT	13,308	7074	27.21	4514	n.a	n.a	n.a	n.a
NL	24,625	12,551	24.33	5716	20,676	12,557	26.11	5333
NO	42,583	19,481	21.12	2466	31,392	41,109	23.90	3470
PL	6213	4508	32.26	15,643	3229	2744	37.33	21,647
PT	11,120	8120	33.30	5618	10,280	8992	37.20	5623
RO	2597	1720	33.44	7636	n.a	n.a	n.a	n.a
SE	26,077	10,900	21.37	2791	20,293	9090	21.96	2973
SI	13,676	6166	23.09	4675	10,122	4656	23.47	4395
SK	7493	3884	26.10	7196	3329	2053	26.67	7554
UK	23,364	21,381	32.29	5789	26,047	22,147	33.05	8588

Mean income and income inequality are computed on the distribution of equivalized household disposable income, variable HX090 of EU-SILC 2005 and 2011. Income inequality is computed using the Gini Index

The outcome variable of interest here is represented by tertiary educational attainment of individuals: a binary variable indicating whether or not the individual has obtained a tertiary education degree. We look at completion of tertiary education since it is well known that individual circumstances play an important role in accounting for (access to and) completion of tertiary education. Higher completion rates, coupled with a stronger redistributive role of the welfare state and an improved quality of the institutional system, should foster intergenerational mobility and help achieving higher equity in wage, income, or consumption. Obtaining a tertiary education degree increases the chances of having access to better and highly remunerated jobs and, on average, to enjoy higher standards of living (compared to those enjoyed by less educated individuals). Certainly, since education can be partly conceived as a positional good, other characteristics, such as the prestige and location of a given institution, may come into play and become key determinants of future jobs and earnings. However, this is especially the case of countries with very high tertiary education completion rates. Given that our analysis is restricted to the European context in the period 2005–2011, with countries characterized by different levels of universality in education, the binary variable “completion of tertiary education” is adequate to capture the measure of the individual advantage^21^ in a comparative setting. In fact, as discussed in Section Measuring Inequality of Opportunity in Tertiary Education, the focus of the analysis on completion of tertiary education produces some important insights, from both a normative and a policy-oriented perspective.

As for the variables capturing circumstances, we use gender, parental education, parental occupation and financial problems when the individual was a teenager.

Parental education is coded into the following five categories: (1) both parents have low or no education; (2) at least one parent has completed medium education, while the other has low or no education; (3) only the father has attained high education; (4) only the mother has attained high education; (5) both parents have attained high education.^22^

Parental occupation is coded into the following five categories: (1) both parents are not working or the information is unknown; (2) at least one parent is a blue collar while the other is not working or the information is unknown; (3) the mother is white collar and father is blue collar or not working/unknown; (4) the father is white collar and the mother is blue collar or not working/unknown; (5) both parents are white collars.^23^

The variable capturing financial problems is coded differently in the two waves since the questions posed to individuals were different. In 2005, the individual was asked how often she experienced financial problems when she was a teenager. We coded it into the following five categories: (1) most of the time; (2) often; (3) occasionally; (4) rarely; (5) never. In 2011, the individual was asked about the financial situation of the family when she was a teenager. We coded this variable again into five categories: (1) vary bad or bad; (2) moderately bad; (3) moderately good; (4) good; (5) very good.^24^ As a consequence of the different coding of the variable capturing financial constraints during teenage years in the two waves—coupled with the fact that such information is missing for some countries in 2005—any across-country comparison over time needs to be interpreted with caution. On the one hand, both variables are good proxies for the financial constraints experienced while a teenager, but, on the other one, the difference in the coding prevent us from making a direct comparison of the estimated coefficients. For this reason we provide a robustness analysis by excluding such variable(s) from the set of individual circumstances.

It is also important to note that our empirical estimates of educational inequality of opportunity are, in each and every case, lower-bound estimates of inequality of opportunity. This caveat applies to all the empirical literature analysing inequality of opportunity. A formal proof of the lower-bound result is contained in Ferreira and Gignoux (2011), but the intuition is straight-forward: the set of circumstances, which is observed empirically, is a strict subset of the set of all circumstance variables that matter in reality—virtually a certainty in all practical applications. None of existing databases is able to register information on the whole set of exogenous factors that can affect any individual outcome, and the values of the inequality of opportunity estimates can only increase as more circumstance variables become observable and are included in the analysis. Moreover, the difference between the estimated and the “true” value of EIOp is exacerbated by the coarse measurement of the observable circumstances, such as parental education and occupation. The use of a limited number of categories to capture each circumstance is the result of both limited information available in the data and the need to find comparable variables across countries and time periods (which, by itself, often requires using coarser measures). Both aspects tend to lower the values of EIOp estimates.

EIOp in tertiary education in 2011 (and 2005) is estimated in each country separately and considering (i) the entire population and (ii) two cohorts: the first cohort is composed by individuals in the age interval 43–60 and the second cohort by individuals in the age interval 25–42.

The variables used in the second part of our analysis—to assess the association existing between EIOp and some country-specific characteristics—are drawn from UNESCO.^25^ In order to minimise the endogeneity risk, we average their values over the five years prior to the survey year (2000–2004 for individuals surveyed in 2005, and 2006–2010 for individuals surveyed in 2011).

## Measuring Inequality of Opportunity in Tertiary Education

In order to obtain (lower-bound) estimates of EIOp, as a first step and separately for each country, we estimate (2) with a probit model relating individual's completion of tertiary education with the set of circumstances.

Table 2 contains the probit estimates for all countries for the 2011 wave. The reference categories are: “female” for gender; “both parents have low or no education” for parental education; “both parents do not work or the information is unknown” for parental occupation; “very good” for the extent of financial problems when a teenager.

**Table 2:** Reduced-form regression of tertiary education achievement on circumstances

Circumstances		AT	BE	BG	CH	CY	CZ	DE	DK
Gender	Male	0.209***	− 0.210***	− 0.506***	0.463***	− 0.0899***	0.0200	0.236***	− 0.208***
		(0.00163)	(0.00135)	(0.00168)	(0.00169)	(0.00498)	(0.0392)	(0.000489)	(0.00226)
Parental Education	M or F at least medium edu	0.209***	0.549***	0.662***	0.332***	0.723***	0.640***	0.380***	0.283***
		(0.00205)	(0.00165)	(0.00203)	(0.00227)	(0.00669)	(0.0505)	(0.000763)	(0.00283)
	F high edu	0.722***	1.070***	1.264***	0.996***	1.129***	1.152***	0.968***	0.755***
		(0.00276)	(0.00254)	(0.00461)	(0.00341)	(0.0123)	(0.0808)	(0.000909)	(0.00446)
	M high edu	0.588***	0.875***	1.309***	0.872***	0.865***	1.267***	0.737***	0.630***
		(0.00624)	(0.00302)	(0.00370)	(0.00781)	(0.0218)	(0.118)	(0.00152)	(0.00411)
	Both high edu	1.163***	1.572***	1.856***	1.157***	1.480***	1.741***	1.365***	0.978***
		(0.00486)	(0.00303)	(0.00387)	(0.00578)	(0.0190)	(0.119)	(0.00122)	(0.00445)
Parental Occupation	M or F at least bc	− 0.0528***	0.196***	0.0240***	− 0.00656**	0.0191***	− 0.0587	− 0.0735***	− 0.0869***
		(0.00249)	(0.00255)	(0.00354)	(0.00304)	(0.00653)	(0.0920)	(0.000939)	(0.00550)
	F wc	0.127***	0.284***	0.217***	0.113***	0.161***	0.273***	− 0.0257***	0.122***
		(0.00302)	(0.00274)	(0.00243)	(0.00325)	(0.0120)	(0.0602)	(0.000971)	(0.00509)
	M wc	0.419***	0.404***	0.248***	0.366***	0.163***	0.112	0.116***	0.236***
		(0.00253)	(0.00259)	(0.00360)	(0.00288)	(0.00802)	(0.0821)	(0.000912)	(0.00541)
	Both white collar	0.490***	0.421***	0.532***	0.432***	0.626***	0.454***	0.125***	0.405***
		(0.00266)	(0.00275)	(0.00243)	(0.00301)	(0.0105)	(0.0684)	(0.000933)	(0.00491)
Financial hardship when teenager	Bad/Very Bad	− 0.164***	− 0.399***	− 0.561***	− 0.0467***	− 0.791***	− 0.102	− 0.154***	− 0.280***
		(0.00388)	(0.00337)	(0.00521)	(0.00410)	(0.0126)	(0.132)	(0.00119)	(0.00545)
	Moderately bad	− 0.121***	− 0.229***	− 0.293***	0.226***	− 0.303***	− 0.0759	0.00387***	− 0.0533***
		(0.00365)	(0.00324)	(0.00405)	(0.00389)	(0.0126)	(0.116)	(0.00109)	(0.00523)
	Moderately good	− 0.0560***	− 0.101***	− 0.105***	0.165***	− 0.234***	− 0.00376	0.00469***	− 0.0564***
		(0.00345)	(0.00259)	(0.00313)	(0.00307)	(0.0116)	(0.109)	(0.000968)	(0.00369)
	Good	− 0.0248***	− 0.0594***	− 0.0730***	0.121***	− 0.289***	− 0.0652	0.0169***	− 0.0627***
		(0.00355)	(0.00248)	(0.00327)	(0.00294)	(0.0117)	(0.110)	(0.000968)	(0.00367)

Circumstances		EE	ES	FI	FR	GR	HR	HU	IE
Gender	Male	− 0.653***	− 0.148***	− 0.446***	− 0.195***	− 0.0811***	− 0.291***	− 0.376***	0.0837***
		(0.00376)	(0.000615)	(0.00175)	(0.000573)	(0.00128)	(0.00254)	(0.00147)	(0.00255)
Parental Education	M or F at least medium edu	0.151***	0.535***	0.329***	0.716***	0.726***	0.578***	0.604***	0.486***
		(0.00500)	(0.00108)	(0.00209)	(0.000876)	(0.00172)	(0.00311)	(0.00178)	(0.00278)
	F high edu	0.462***	0.919***	0.804***	0.914***	1.012***	1.163***	1.297***	1.041***
		(0.00875)	(0.00134)	(0.00341)	(0.00125)	(0.00298)	(0.00548)	(0.00318)	(0.00587)
	M high edu	0.718***	0.820***	0.629***	0.935***	1.532***	0.978***	1.029***	0.975***
		(0.00693)	(0.00255)	(0.00346)	(0.00148)	(0.00682)	(0.00703)	(0.00414)	(0.00645)
	Both high edu	0.879***	1.360***	1.009***	1.428***	1.330***	1.625***	1.574***	1.367***
		(0.00773)	(0.00231)	(0.00347)	(0.00153)	(0.00369)	(0.00689)	(0.00380)	(0.00855)
Parental Occupation	M or F at least bc	− 0.0781***	0.00880***	− 0.265***	− 0.162***	0.133***	0.0264***	− 0.0180***	0.175***
		(0.00735)	(0.00100)	(0.00430)	(0.000941)	(0.00173)	(0.00394)	(0.00243)	(0.00399)
	F wc	0.239***	0.167***	0.00585**	0.0351***	0.0761***	0.218***	0.339***	0.344***
		(0.00526)	(0.00169)	(0.00277)	(0.00101)	(0.00313)	(0.00484)	(0.00215)	(0.00583)
	M wc	0.134***	0.391***	-0.0367***	0.247***	0.371***	0.253***	0.0576***	0.600***
		(0.00852)	(0.00107)	(0.00373)	(0.000936)	(0.00196)	(0.00429)	(0.00289)	(0.00440)
	Both white collar	0.555***	0.388***	-0.0524***	0.418***	0.345***	0.446***	0.516***	0.662***
		(0.00613)	(0.00143)	(0.00253)	(0.000972)	(0.00252)	(0.00450)	(0.00244)	(0.00547)
Financial hardship when teenager	Bad/Very Bad	− 0.229***	− 0.233***	− 0.359***	− 0.143***	− 0.374***	− 0.175***	− 0.0999***	− 0.324***
		(0.0153)	(0.00243)	(0.00539)	(0.00167)	(0.00337)	(0.00570)	(0.00535)	(0.00653)
	Moderately bad	− 0.283***	− 0.0310***	− 0.165***	0.0824***	− 0.280***	− 0.117***	0.0445***	− 0.278***
		(0.0139)	(0.00234)	(0.00487)	(0.00155)	(0.00321)	(0.00567)	(0.00499)	(0.00629)
	Moderately good	− 0.141***	0.252***	− 0.0920***	0.213***	− 0.0913***	− 0.0190***	0.167***	− 0.141***
		(0.0134)	(0.00225)	(0.00450)	(0.00142)	(0.00291)	(0.00510)	(0.00482)	(0.00573)
	Good	− 0.0492***	0.382***	− 0.0864***	0.0964***	− 0.123***	− 0.109***	0.146***	− 0.0733***
		(0.0137)	(0.00225)	(0.00459)	(0.00145)	(0.00300)	(0.00511)	(0.00493)	(0.00591)

Circumstances		IS	IT	LT	LU	LV	MT	NL	NO
Gender	Male	− 0.484***	− 0.151***	− 0.394***	0.101***	− 0.592***	− 0.0147*	0.149***	− 0.245***
		(0.00775)	(0.000651)	(0.00247)	(0.00855)	(0.00300)	(0.00764)	(0.00104)	(0.00187)
Parental Education	M or F at least medium edu	0.391***	0.662***	0.654***	0.356***	0.361***	0.513***	0.375***	0.347***
		(0.00931)	(0.000829)	(0.00292)	(0.00986)	(0.00374)	(0.00886)	(0.00127)	(0.00265)
	F high edu	0.849***	1.316***	1.192***	1.101***	0.801***	0.881***	0.818***	0.741***
		(0.0151)	(0.00172)	(0.00663)	(0.0190)	(0.00767)	(0.0158)	(0.00174)	(0.00345)
	M high edu	0.625***	1.193***	1.179***	0.823***	0.854***	1.176***	1.201***	0.642***
		(0.0217)	(0.00255)	(0.00542)	(0.0309)	(0.00580)	(0.0334)	(0.00319)	(0.00416)
	Both high edu	1.459***	1.695***	1.749***	1.905***	1.182***	0.967***	1.452***	1.263***
		(0.0237)	(0.00258)	(0.00620)	(0.0345)	(0.00766)	(0.0254)	(0.00250)	(0.00381)
Parental Occupation	M or F at least bc	− 0.195***	− 0.0361***	− 0.0737***	0.0113	− 0.0708***	0.0173	0.0688***	− 0.0509***
		(0.0143)	(0.00104)	(0.00445)	(0.0141)	(0.00540)	(0.0192)	(0.00203)	(0.00395)
	F wc	0.121***	0.309***	0.195***	0.152***	0.275***	0.476***	0.220***	0.214***
		(0.0128)	(0.00147)	(0.00356)	(0.0178)	(0.00406)	(0.0290)	(0.00261)	(0.00348)
	M wc	0.130***	0.279***	− 0.0377***	0.430***	0.505***	0.311***	0.427***	0.242***
		(0.0137)	(0.00111)	(0.00480)	(0.0148)	(0.00666)	(0.0190)	(0.00199)	(0.00372)
	Both white collar	0.235***	0.456***	0.210***	0.647***	0.503***	0.731***	0.486***	0.467***
		(0.0130)	(0.00133)	(0.00370)	(0.0174)	(0.00541)	(0.0244)	(0.00220)	(0.00346)
Financial hardship when teenager	Bad/Very Bad	− 0.281***	− 0.573***	− 0.277***	− 0.438***	− 0.0926***	− 0.269***	0.0311***	− 0.273***
		(0.0196)	(0.00261)	(0.00881)	(0.0263)	(0.00848)	(0.0320)	(0.00293)	(0.00585)
	Moderately bad	0.117***	− 0.310***	− 0.180***	− 0.422***	− 0.0752***	0.00843	0.189***	0.248***
		(0.0172)	(0.00241)	(0.00864)	(0.0236)	(0.00775)	(0.0305)	(0.00255)	(0.00458)
	Moderately good	0.0171	− 0.0910***	0.0245***	0.00993	0.0948***	0.223***	0.168***	0.162***
		(0.0150)	(0.00227)	(0.00808)	(0.0187)	(0.00700)	(0.0292)	(0.00205)	(0.00372)
	Good	0.178***	− 0.0441***	0.0397***	0.0290	0.00931	0.342***	0.0141***	0.117***
		(0.0161)	(0.00233)	(0.00817)	(0.0183)	(0.00724)	(0.0290)	(0.00191)	(0.00364)

Circumstances		PL	PT	RO	SE	SI	SK	UK
Gender	Male	− 0.287***	− 0.498***	− 0.0870***	− 0.434***	− 0.369***	− 0.159***	− 0.0417***
		(0.000702)	(0.00165)	(0.00105)	(0.00148)	(0.00299)	(0.00186)	(0.000589)
Parental Education	M or F at least medium edu	0.666***	0.581***	0.979***	0.348***	0.381***	0.478***	0.335***
		(0.000892)	(0.00322)	(0.00135)	(0.00179)	(0.00409)	(0.00259)	(0.000715)
	F high edu	1.241***	1.327***	1.689***	0.869***	0.765***	1.123***	0.810***
		(0.00194)	(0.00542)	(0.00336)	(0.00310)	(0.00683)	(0.00456)	(0.00116)
	M high edu	1.338***	0.825***	2.147***	0.846***	0.558***	0.951***	0.584***
		(0.00220)	(0.00560)	(0.00785)	(0.00249)	(0.00788)	(0.00676)	(0.00119)
	Both high edu	1.508***	1.385***	1.975***	1.330***	0.996***	1.584***	1.079***
		(0.00186)	(0.00578)	(0.00378)	(0.00299)	(0.00811)	(0.00564)	(0.00138)
Parental Occupation	M or F at least bc	− 0.0286***	0.182***	0.0178***	0.725***	− 0.0685***	− 0.0358***	0.145***
		(0.00113)	(0.00222)	(0.00151)	(0.00835)	(0.00461)	(0.00388)	(0.00108)
	F wc	0.441***	0.336***	0.329***	0.684***	0.246***	0.359***	0.240***
		(0.00103)	(0.00328)	(0.00184)	(0.00699)	(0.00485)	(0.00276)	(0.00101)
	M wc	0.304***	0.462***	-0.0208***	0.746***	0.180***	0.335***	0.288***
		(0.00124)	(0.00242)	(0.00183)	(0.00677)	(0.00571)	(0.00382)	(0.00103)
	Both white collar	0.651***	0.841***	0.366***	0.882***	0.394***	0.696***	0.593***
		(0.00108)	(0.00287)	(0.00154)	(0.00656)	(0.00534)	(0.00298)	(0.000999)
Financial hardship when teenager	Bad/Very Bad	− 0.179***	− 0.259***	− 0.542***	− 0.0946***	0.286***	− 0.286***	− 0.115***
		(0.00235)	(0.00749)	(0.00417)	(0.00330)	(0.00881)	(0.00567)	(0.00161)
	Moderately bad	− 0.0773***	0.255***	− 0.349***	0.0152***	0.393***	− 0.0983***	0.0960***
		(0.00218)	(0.00735)	(0.00409)	(0.00318)	(0.00830)	(0.00487)	(0.00145)
	Moderately good	0.123***	0.497***	− 0.273***	0.0685***	0.385***	− 0.0380***	0.113***
		(0.00201)	(0.00715)	(0.00396)	(0.00238)	(0.00823)	(0.00455)	(0.00132)
	Good	0.179***	0.649***	− 0.176***	0.0416***	0.300***	− 0.0792***	0.157***
		(0.00200)	(0.00722)	(0.00408)	(0.00227)	(0.00871)	(0.00458)	(0.00135)

The reference categories are: “female” for gender; “both parents have low or no education” for parental education; “both parents do not work or the information is unknown” for parental occupation; “very good” for the extent of financial problems when a teenager. Standard errors in parentheses

***p < 0.01, **p < 0.05, *p < 0.1

For the sake of clarity, only the estimates for 2011 are discussed in the main text, as in most of the cases they are similar to the estimates for 2005 (which are presented in the Table 7 in Annex 1). As this is a reduced-form equation, our estimates cannot be interpreted causally. In fact, the estimated coefficients capture both the direct and the indirect effects of circumstances on the outcome variable (see Ferreira & Gignoux, 2011).

Almost all the coefficients have the expected sign and are statistically significant. Relative to the reference group (students having both parents with low or no education), students whose parents have completed secondary or tertiary education have a higher probability of completing tertiary education. Tertiary education attainment and parental occupation are also positively correlated. Results show that, with respect to the omitted category (category 1 listed above: neither of the parents is working or the information is unknown), individuals who have at least one parent employed in a white collar job have higher probabilities of completing tertiary education. Notice that the coefficients for parental occupation are usually lower than those for parental education.

“Financial hardship when teenager” is negatively correlated with the probability that descendants attain tertiary education. Gender is also significant in determining the probability of completing tertiary education. However, differently from the previous circumstances, the sign of its impact cannot be stylized as it varies across countries.

**Table 3:** Inequality of opportunity for tertiary education and contribution of each circumstance, 2011

Country	EIOp	Gender	Parental education	Parental occupation	Childhood financial hardship
AT	23.77	9.52	32.75	43.38	14.35
BE	22.63	5.17	53.17	29.63	12.03
BG	35.71	15.83	43.41	31.16	9.60
CH	18.62	28.79	32.59	32.27	6.35
CY	27.14	1.96	47.22	30.95	19.87
CZ	36.58	0.29	56.23	34.02	9.46
DE	20.52	11.43	55.94	22.28	10.35
DK	18.37	9.07	47.24	32.94	10.76
EE	22.08	30.49	35.74	25.94	7.83
ES	20.90	4.84	35.09	38.11	21.96
FI	15.32	29.86	48.69	9.66	11.73
FR	24.49	4.74	50.21	33.10	11.95
GR	25.22	1.22	52.55	30.61	15.63
HR	33.33	8.39	45.08	32.29	14.24
HU	36.31	8.97	45.43	33.46	12.41
IE	20.05	3.82	44.85	36.95	14.39
IS	19.25	32.24	34.71	21.45	11.60
IT	35.41	3.50	45.83	34.30	16.37
LT	26.38	10.35	49.94	26.25	13.46
LU	27.45	3.50	33.38	44.36	18.77
LV	25.72	28.21	32.47	30.35	8.97
MT	26.64	0.37	46.87	32.30	20.45
NL	20.86	4.88	50.48	38.48	5.83
NO	19.26	8.55	47.90	36.05	7.51
PL	32.23	7.72	38.85	38.64	14.79
PT	38.05	13.73	21.06	34.72	30.49
RO	38.43	1.55	52.64	29.82	15.98
SE	18.91	25.77	55.88	14.48	3.86
SI	23.25	14.32	40.32	33.77	11.58
SK	28.46	4.39	35.13	45.93	14.55
UK	16.95	0.85	52.57	34.61	11.94

The estimates of lower-bound EIOp in tertiary education for 2011 (those for 2005 are reported in Table 8 in Annex 1) show large across-country variation, from a minimum of 15.32 in Finland to a maximum of 38.43 in Romania. On average, EIOp is lowest in Northern European countries as compared to Mediterranean and Eastern European countries (a similar picture emerges for 2005).

The results of the decomposition by source of inequality of opportunity are very robust to the survey year (see Table 3 and Table 8 in Annex 1). In all cases, parental education and parental occupation contribute most to EIOp. Financial problems experienced by the family when the individual was a teenager is also a relevant circumstance, but much less than parental education and occupation. As explained in Section Data, the measurement of financial problems differs between 2005 and 2011, hence a strict numerical comparisons should be avoided. Notwithstanding, the inclusion of this circumstance is insightful and reveals that, on average, financial constraints play a non-negligible role in determining the distribution of tertiary education opportunities (although the most relevant circumstances remain parental education and occupation). Gender, instead, appears to contribute only slightly to EIOp in tertiary education, at least relatively to the other circumstances. At the same time, we notice some relevant across-country variability in the role of the two factors that appear to contribute less. Financial distress when teenager appears quite relevant in Portugal, Spain, Cyprus, and Malta. Among Eastern countries, only Romania and Slovakia show high values for the (relative) contribution of financial constraints. When it comes to gender, Island, Estonia, Sweden, Finland, Switzerland, and Latvia are the countries where the relative contribution of this circumstance is higher. However, this should not induce us to conclude that some sort of gender discrimination in the achievement of tertiary education is present in these countries. Since we are capturing relative contributions, as the relative importance of parental education and occupation is reduced in these countries, the values for the contributions from the other determinants are mechanically increased. As for the other countries, the lower contribution of gender to EIOp (relative to the other circumstances) also needs to be interpreted with caution. For instance, gender could significantly affect the choice of the field in which an individual studies^26^ and hence the likelihood of finding a job, its quality and the wage. However, when focusing solely on the likelihood of completing tertiary education, we do not find any significant difference between men and women.

**Table 4:** Inequality of opportunity for tertiary education by cohort and difference in EIOp and in the contribution of each circumstance between the two cohorts (value for the younger cohort minus value for the older cohort), 2011

			Difference in EIOp and in the role of circumstances between younger and older cohort			
Country	Older cohort	Younger cohort	EIOp	Gender	Parental education and occupation	Childhood financial hardship
AT	24,66	22,75	− 1,91	− 16,57	11,66	4,92
BE	24,83	19,93	− 4,9	14,15	− 13,49	− 0,81
BG	36,05	35,76	− 0,9	11,51	− 9,16	− 2,35
CH	21,34	15,85	− 5,49	-18,66	21,35	− 2,69
CY	25,79	18,53	− 7,26	2,25	16,81	− 19,06
CZ	40,14	31,76	− 8,38	-0,05	2,42	− 2,37
DE	21,1	19,49	− 1,61	-14,17	7,57	6,6
DK	16,09	17,08	0,99	9,99	− 5,2	− 4,73
EE	22,14	22,24	0,1	9,13	− 5,45	− 5,68
ES	24,53	16,8	− 7,73	13,56	− 10,82	− 2,73
FI	15,77	14,89	− 0,88	37,64	− 30,49	− 7,15
FR	26,23	19,88	− 6,35	5,29	0,76	− 6,04
GR	22,7	25,08	2,38	0,64	10,93	− 11,48
HR	34,22	30,16	− 4,06	22,76	− 9,76	− 8,06
HU	35,57	35	− 0,57	6,88	− 3,56	− 3,31
IE	22,05	14,33	− 7,72	-0,47	− 8,53	8,99
IS	20,21	17,98	− 2,23	-0,62	− 12,65	13,26
IT	35,94	32,85	− 3,09	8,04	− 3,91	− 4,14
LT	26,43	21,19	− 5,24	5,99	− 1,89	− 4,11
LU	29,86	23,6	− 6,26	-7,79	− 1,25	9,04
LV	26,91	23,73	− 3,18	9,68	− 9,73	0,05
MT	35,33	25,39	− 9,94	-3,71	6,4	− 2,69
NL	21,28	18,16	− 3,12	-10,91	8,17	1,74
NO	21,43	17,01	− 4,42	11,45	− 11,51	0,06
PL	36,66	23,76	− 12,9	9,57	− 7,77	− 1,8
PT	41,28	34,16	− 7,12	11,46	2,83	− 14,29
RO	30,2	39,38	9,18	2,2	7,34	− 9,55
SE	15,87	17,38	1,51	-17,15	19,08	− 1,94
SI	25,65	20,27	− 5,38	20,38	− 14,24	− 6,14
SK	33,17	23,41	− 9,76	14,57	− 8,63	− 5,94
UK	18,53	14,8	− 3,73	2,79	− 4,03	1,24

**Fig. 1:**
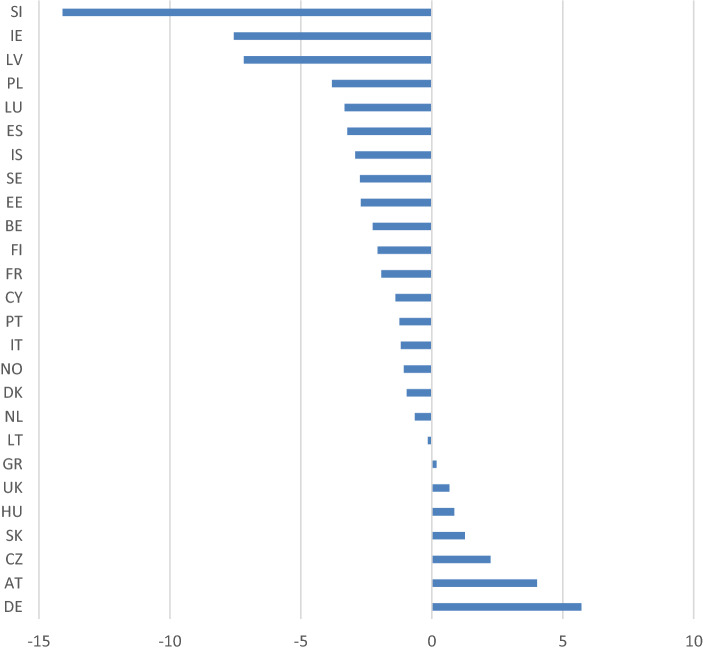
EIOp in tertiary education: variation from 2005 to 2011

The changes in EIOp between 2005 and 2011 also vary across countries (see Fig. 1).

Worth noticing is the considerable increase in EIOp in both Germany (from 14.79 to 20.52) and Austria (from 17.08 to 23.77). These changes, however, could reflect the introduction of the “Financial problem” circumstance, which was not available for the two countries in 2005 (it became available only in 2011). At the other extreme, we observe a decrease in EIOp in Slovenia (from 36.48 to 23.25), Ireland (from 26.3 to 20.05) and Latvia (from 32.57 to 25.72).

When assessing EIOp, we have to keep in mind that cohort-specific effects might play an important role, since tertiary educational attainment has a clear age-related dimension (and it is normally over by age 24). For instance, reforms affecting participation to tertiary education—for instance by changing its rules and costs—only affect individuals that at the time of the reform have not decided yet whether to attend university or not. Analogously, technological progress (and its impact on the labour market returns from tertiary education) might affect more some cohorts than others. Hence, time trends can hide relevant across-cohort heterogeneity.

The disaggregation by cohort reported in Table 4 for the 2011 wave reveals that the oldest cohort (43–60) experiences a higher degree of EIOp almost everywhere, with the exception of Greece (showing slightly higher EIOp for the youngest cohort), and Romania (where this difference becomes dramatic: about 9 percentage points). There are almost no across-cohort differences, instead, in Austria, Bulgaria, Germany, Denmark, Estonia, Hungary and Sweden. For the remaining countries, EIOp for the younger cohort is much lower than the one faced by the older cohort, especially in Eastern countries, such as, Poland, Slovakia and Czech Republic. Sizable differences also arise in Cyprus, Malta, Portugal, and Ireland.

**Table 5:** Pair-wise correlations—26 countries—EIOp measured in 2005 and 2011—characteristics measured by average of previous five years

Institutional variables	EIOp
Gross enrolment rate in tertiary education	− 0.3181**
Share of individuals aged 25 and older with at least upper secondary education	− 0.2406*
Share of individuals aged 15–25 enrolled in vocational studies	0.0216
Share of all secondary education student enrolled in general programmes	− 0.0319
Government expenditure on education (% GDP)	− 0.4613***
Government expenditure on tertiary education (% GDP)	− 0.5130***
Government expenditure on education (% of total government expenditure)	− 0.3813***
Government expenditure on tertiary education (% of total government expenditure)	− 0.5496***
Inbound mobility, tertiary	− 0.1600
Outbound mobility, tertiary	0.0908
Students—teachers ratio, tertiary	0.3115**
Employment rate tertiary/secondary	0.3652***
Unemployment rate tertiary/secondary	− 0.2307*
Relative earnings: tertiary/upper secondary	0.5571***

***p < 0.01, **p < 0.05, *p < 0.1.

Next, we analyse the across-cohort changes in the sources of inequality. The goal here is to understand the changes in the relative importance of the various factors (gender, parental education and occupation, financial constraints when a teenager) in accounting for the observed patterns.

When making cohort comparisons, it is instructive to look at Table 4 reporting the difference (in percentage points) in the contribution of each circumstance between the younger and the older cohort.^27^

Interestingly, gender becomes relatively less important for the younger cohort in Austria, Switzerland, Germany, Netherland, and Sweden; it is quite stable in Malta, Iceland, Check Republic, Greece, UK, Romania, Ireland, and Cyprus. It becomes more important elsewhere. The contribution of financial hardship is relatively stable or slightly decreasing, with the notable exceptions of Ireland and Iceland on one side—financial problems being relatively more important in the younger cohort—and Portugal, Romania, and Greece on the other side—financial problems being relatively less important in the younger cohort. Last, the change in the contribution of parental education and occupation between the two cohorts is widely variable across countries (the change from the older cohort to the younger one ranges between + 21 percentage points in Switzerland to -30 percentage points in Finland).

For completeness, we also run several robustness checks.

The first concerns the choice of including financial hardship as a circumstance: our main results could be affected by the lack of data on such variable for some countries in 2005. Table 9 in Annex 2 reports the estimates of EIOp in 2005 and 2011 excluding “Financial hardship when teenager” from the set of circumstances. The estimated variation of EIOp between 2005 and 2011—for Germany and Austria, but also for the other countries for which information on financial constraints is available only in 2011—is slightly reduced. Given the small (relative) contribution of this variable to overall EIOp, its exclusion does not significantly affect the main results. The country ranking is quite stable (with Romania and Finland being respectively the countries with the highest and lowest EIOp values). A noticeable variation can be seen only for two countries: Portugal—whose EIOp change over time goes from -1.29 percentage points (when financial problems during childhood is included in the set of circumstances) to 2.9—and Hungary—whose EIOp change over time goes from 0.86 to 4.65.

The second robustness check concerns the choice of the specific cohort partition adopted in the main analysis, justified by the need to compare cohorts with an equal age interval. In Annex 3 we report the EIOp estimates in 2011 for four alternative cohort partitions. The first partition aims at identifying differences between young individuals, possibly at the beginning of their job career and the rest of the population (25–35 vs. 35–60, Table 10). The second partition, by contrast, aims at identifying differences between older individuals, possibly at the end of their job career, and the rest of the population (25–50 vs. 50–60, Table 10). Last, we estimate EIOp by partitioning the population into three cohorts (25–35 vs. 35–45 vs. 45–60 and 25–40 vs. 40–50 vs. 50–60 in Table 11). The results are consistent with those of Table 4. There are only two exceptions to this. For Iceland and Estonia we find that the youngest cohort tends to exhibit the highest degree on EIOp compared with the other cohorts (see Table 10). When focusing only on a three cohorts partition, it can be observed again that for the majority of countries EIOp is higher among older cohorts. One exception is Romania, a country in which EIOp decreases for the older cohort (see also Table 4). The other exceptions are Estonia and Croatia, where EIOp first increases and then decreases with the age of the cohort (inverse-U shape, see Table 11).

The third robustness check concerns the treatment of the migrant population. The main model (Table 3) uses only observations on natives. When considering the extension to migrants, it is important to recognize that some of them might have migrated while adults, other when still young and others might be second-generation migrants. Given the focus on country-specific estimates of EIOp, which are also related to country-specific characteristics and institutions (see Section Exploring Drivers of EIOp), in the main model it is preferable to exclude (resident) individuals who moved to the destination country only as adults (possibly after graduation in their birth country). However, for 2011 (the year of immigration is available only for 2011), it is also possible to estimate EIOp using the whole sample of residents (i.e. natives plus migrants) with the exclusion of adult immigrants (all those who were older than 25 when migrated). The results are reported in Table 12 in Annex 4 and do not show relevant variation with those reported in Table 3.

Last, we ask if and how the estimates of EIOp change when—as an additional circumstance—we consider whether an individual is second generation migrant (in this case the sample is made up by natives and second generations migrants only). Again this information in only available for 2011. As indicated in Table 13 in Annex 5, the main characteristics highlighted in Table 3 are confirmed, mainly because this circumstance plays the least relevant role relatively to all the others.

## Exploring Drivers of EIOp

**Fig. 2:**
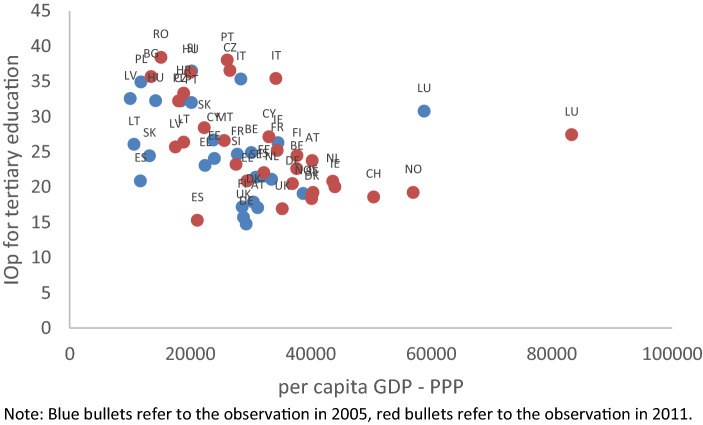
EIOp and GDP per capita. Blue bullets refer to the observation in 2005, red bullets refer to the observation in 2011

To investigate the drivers of inequality of opportunity in tertiary education, we relate the EIOp estimates described in the previous section to country-specific characteristics capturing features of the education system or of the labour market that have a more direct relationship with the costs and benefits of tertiary education. But before doing so it is interesting to note that the relationship between EIOp estimates and real GDP per capita (in PPP current international $) is negative (− 0.39) and highly significant (p-value equal to 0.0027). In Fig. 2, by visual inspection, we can identify two clusters of countries. The first cluster is made of Eastern European countries, characterized by higher EIOp and lower per capita GDP; most of them, in fact, have EIOp levels that are above those that would be associated with their levels of development. Lithuania and Estonia are some exceptions, as their level of development is associated to a relatively low EIOp. The second cluster is made by Western countries with middle/high level of development and middle/low level of EIOp. Exceptions are Italy and Luxembourg, exhibiting very high EIOp values, relative to their per capita GDP.

These results, indicating that—on average—richer countries tend to have lower levels of EIOp, are consistent with the hypothesis that there is no trade-off between equality of opportunity in tertiary education and growth (see Marrero & Rodríguez, 2013). However, they also show that the same (or very similar) values of per-capita GDP are associated with different estimates of EIOp, possibly as a result of country-specific (and possibly time-varying) features, whose role is explored in the next paragraphs.

Table 5 reports the result of the pair-wise correlations between EIOp and all the indicators capturing specific features of the education system and or labour markets of the different countries.

**Table 6:** Pooled OLS and FE estimates

	Dependent variable: EIOp			
Variables	Pooled OLS	FE	Pooled OLS	FE
Government expenditure on tertiary education	− 6.83*** (2.89)	− 4.95*** (2.64)	− 9.49*** (2.20)	− 5.25*** (2.61)
Students—teachers ratio in tertiary education	0.39***	0.51***	0.34***	0.53***
	(0.16)	(0.16)	(0.15)	(0.16)
Gross enrolment rate in tertiary education	− 0.12** (0.06)	− 0.07 (0.05)	n.a	n.a
Share of individuals aged 25 and older with at least upper secondary education	n.a	n.a	− 0.03 (0.048)	− 0.02 (0.05)
Unmployment rate tertiary/secondary	− 1.89 (6.34)	− 0.44 (6.92)	− 2.53 (6.33)	− 1.74 (6.77))
Constant	36.62*** (5.69)	29.36*** (5.47)	35.77*** (6.17)	27.33*** (5.84)
Observations	46	46	43	43
R-squared Wald-chi squared Prob > chisquared	0.36 n.a n.a	n.a 18.50 0.001	0.38 n.a n.a	n.a 17.74 0.001
Number of countries	27	27	27	27

Standard errors in parentheses. Significance: for Pooled OLS: ***p < 0.01, **p < 0.05, *p < 0.1; for FE: ***z < 0.01, **z < 0.05, *z < 0.1

Several cases are worth noting. The correlation between EIOp and the gross enrolment rate in tertiary education is negative, which does not support the “competition hypothesis” (see Triventi, 2013,28), according to which, as the number of (potential) competitors in education (and, subsequently in the labour market) rises, students from a more advantaged background (the largest driver of EIOp) tend to be more successful in completing higher education. On the other hand, higher shares of individuals aged 25 and older who have at least completed secondary education are negatively correlated with EIOp, in line with the hypothesis that increased completion of secondary education also increases equality of opportunity in tertiary education. Finally, higher shares of 15–25 year old students enrolled in vocational education tend to be associated with higher levels of EIOp, but the coefficient is not statistically significant.^29^ This result indicate that vocational education, per se (i.e. without early tracking), does not necessarily lead to higher inequality of opportunity in higher education.

The amount of public spending allocated to education is significantly and negatively correlated with EIOp irrespective of the variable used. Similarly, public spending on tertiary education (either in percentage of GDP or in percentage of total government expenditure) is strongly negatively correlated with EIOp. Both results are consistent with the hypothesis that additional investments along the entire educational ladder improve equality of opportunity also in the last stage.

The correlation analysis with respect to the quality variables generates ambiguous results. When outbound and inbound mobility are used to proxy quality, they have the expected (and opposite) signs. However, given that in both cases the coefficients are small and not statistically significant, we do not find any robust association between international (outbound and inbound) mobility and EIOp. However, when we proxy quality with the students/teacher ratio in tertiary education, we find that the latter is significantly and positively correlated with EIOp.

Last, we look at the association between EIOp and the economic benefits from tertiary education achievement, as proxied by (i) the relative employment rate of individuals with tertiary education, (ii) the relative unemployment rate of individuals with tertiary education (iii) relative earnings (in all three cases relative to individuals with only secondary education). Results indicate that the relative employment rate and the relative earnings of tertiary graduates are positively and significantly correlated with EIOp, while a negative correlation occurs for the relative unemployment rate. This is an interesting result as it indicates that policies directed at improving equality of opportunity might be particularly needed when the labour market signals that returns to tertiary education are higher.

As a further step, these correlations are forced to more stringent tests using multi-variable regression analysis and exploiting the presence of observation in two points in time (2005 and 2011). Two different model specifications are run: a pooled OLS regression and a fixed effect model (FE), which would be appropriate in case of time-invariant and country specific unobservable factors. We select only the variables that are significantly correlated with EIOp (see Table 5) and for which we have a relatively high number of observation (e.g. for the relative unemployment rate we have roughly twice the number of observations available for the relative wage). Besides, some variables are highly correlated among themselves^30^ and hence only one of them can be used in the regression. It is important to bear in mind that our analysis does not try to estimate causal impacts. Therefore, the coefficients that are presented below (as well as the coefficients reported in Table 6) must be interpreted as simple correlations. Nevertheless, the availability of two observations for most of the countries allows us to increase the precision of our estimates. The results of our estimations are reported in Table 3. It is evident that not all correlations that are highly significant in the pair-wise correlation test are robust to the regression analysis. Public expenditure in tertiary education (measured in percentage of GDP^31^) is significantly and negatively correlated with EIOp in all specifications, indicating that equality of opportunities in tertiary education respond positively to public investments. Students/teacher ratio in tertiary education is also statistically significant in all the models tested, indicating that—ceteris paribus—a reduction of the number of students per teacher (considered as a proxy for the quality of education) tends to be associated with lower EIOp. The gross enrolment rate in tertiary education has a significant negative association with EIOp, but only in the pooled OLS estimation. By contrast, the negative correlation between EIOp and the share of individuals aged 25 or older with at least secondary is never significant. Finally, the variable used to proxy the incentives to achieve tertiary education (i.e. the relative unemployment rate^32^) always enters with a negative but not significant coefficient.

**Fig. 3:**
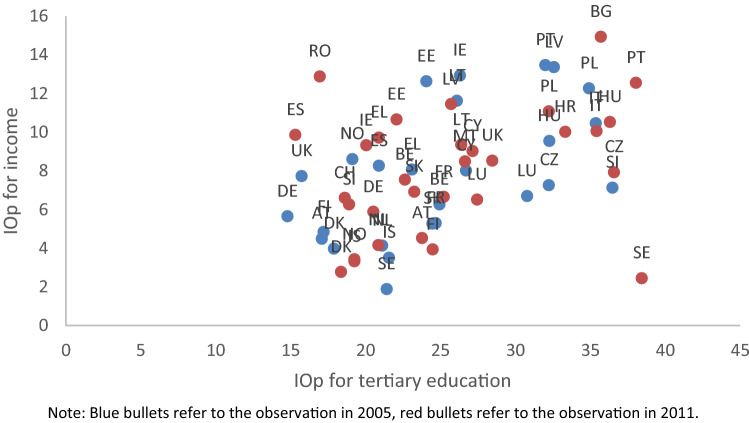
Inequality of opportunity in tertiary education and inequality of opportunity in income, 2005 and 2011. Blue bullets refer to the observation in 2005, red bullets refer to the observation in 2011

As a last step, Fig. 3 plots for each country the combination of inequality of opportunity for tertiary education (EIOp) and inequality of opportunity for income (IOp). There is a clear direct and positive relation between the two dimensions, with a correlation coefficient of 0.4296. Countries with higher inequality of opportunity for tertiary education are also characterized by higher inequality of opportunity for income. In particular, by visual inspection, we can identify three clusters.

The first is composed by countries with high level of both types of inequality, mostly Eastern-European countries, but it includes also Italy and Portugal. The second cluster encompasses countries with average levels of both EIOp and income IOp, and it is made up of Mediterranean and Eastern countries. The third cluster is made of countries with low level of both EIOp and income IOp, encompassing only Northern-European countries. We argue that providing equal opportunities for tertiary education attainment also equalizes opportunities for income. Of course, there are other channels that operate and affect inequality of opportunity for income (see discussion in Sect. 3), but granting equity at the educational stage would increase the probability of facing more equality of opportunity in later stages of life.

The implication that increasing equality of opportunity in (tertiary) education leads to higher equality of opportunity in income is not only valid in the context of the single individual life-cycle. In fact, because of the strong effect that the educational attainment of parents tends to exert on that of their offsprings, increasing educational opportunities for one generation will indirectly benefit future generations as well, in terms of both education and income opportunities.^33^

## Discussion

A growing literature in the field of normative economics looks at the different factors generating inter-individual disparities, distinguishing between fair inequality, that is, inequality that is the result of differences in individuals’ effort, and unfair inequality, that is, inequality caused by factors outside the sphere of the individual responsibility. According to the EOp paradigm, a society is to be considered equitable if "opportunities", rather than outcomes (as in the more traditional welfarist approach), are equally distributed. The opportunity egalitarian perspective is especially relevant when the focus is on education, which is an important determinant of future earning capacity of individuals and, more generally, of their future well-being.

In this study, the EOp framework has been applied to the measurement of inequality of opportunity in tertiary education attainment (EIOp) in Europe, using the EU-SILC database for two survey years, 2005 and 2011. The results, robust to the time period considered, reveal that the (lower-bound) estimates of EIOp are lowest in Northern European countries and highest in Mediterranean and Eastern European countries. The disaggregation by circumstance shows that parental education and parental occupation are the most important factors contributing to EIOp. This points to socio-economic background as the most relevant driver of EIOp (we also note some relevant across-country variation), with primary and secondary effects on educational achievement (primary factors are those that affect academic achievement, while secondary effects capture the role of social background on educational choices, net of academic performance^34^; Boudon, 1974).

Policies directed at improving equality of opportunity in higher education should be focused on reducing the importance of parental background in determining access to (and completion of) tertiary education, as well as on reducing barriers to entry to the more prestigious institutions for those with a disadvantaged background. At the same time it is clear that participation to (and completion of) tertiary education is just the last stage of the educational career, and it depends on all the previous stages.

The interpretation of our results is conditional on some *caveats*. The first *caveat*, as largely discussed in the paper, concerns the set of circumstances and their construction, and it implies that what we observe and describe here are only lower-bound estimates of EIOp. Nevertheless, this *caveat* does not reduce the relevance of our results, which prove to be robust to a variety of circumstance choices.

A second *caveat* relates to the inequality of opportunity approach adopted in this paper. The literature has developed two approaches for the measurement of opportunity inequality, namely the ex-ante and the ex-post approach. According to the ex-ante approach, there is equality of opportunity if the set of opportunities is the same for all individuals, regardless of their circumstances. According to the ex-post approach, there is equality of opportunity if and only if all those who exert the same effort end up with the same outcome. They express different and sometimes conflicting views on equality of opportunity, reflected in the different estimates (and rankings) that they generate (Fleurbaey and Peragine 2013). In addition, their informational requirements are quite different: while for the ex-ante approach one needs to observe the individual outcome and the set of circumstances, for the ex-post approach a measure of individual effort is also required. Since a satisfactory measure of effort is often unavailable, most empirical applications follow the ex-ante approach, as it is done in this study.

These two *caveats* might represent the departing points for future research. Our work could be extended by increasing the set of circumstances and by adopting the ex-post approach to the measurement of EIOp. Both extensions, however, imply that one should opt for using “richer” datasets. Additional insights could come from the evaluation of the impact on EIOp of macroeconomic shocks, such as the crisis generated by the Covid-19 pandemic.

Without the presumption of being exhaustive, below we discuss a set of policies that could improve equality of opportunity in tertiary education.

First, there are policies directed at improving quality for all at the stage of compulsory and pre-school education (including early childhood education and care, which has been shown to produce long-term effect: see García et al., 2016; Heckman et al., 2010; Heckman, 2006). A crucial role here is played by the financing of public compulsory education (general taxes vs property taxes), and by the availability of private education, its costs and the subsidies it receives (directly and indirectly) from the public sector. The extent to which households can select the (public) school for their children is also important, as more affluent parents tend to be more informed about “school quality” and hence take more advantage of “freedom of choice”. Investment in public school (including ICT) infrastructures and in teachers’ professional development (including the use of digital technologies for teaching and learning) are essential to reduce the gaps that exist across public schools and between public and private schools.^35^ Resources should also be devoted to reinforce the learning of more disadvantaged students, who often find a less supportive environment at home. Long summer holidays have also been shown to be a factor contributing to increasing the socio-economic gradient in compulsory education (Stewart et al, 2018).

Concerning upper-secondary school, tracking has been shown to increase educational socio-economic inequalities (Hanushek & Wößmann, 2006; Strello et al., 2021; Volante et al., 2019) for at least three main reasons. First, students in lower tracks tend to have a negative attitude towards schooling, and low expectations on educational achievement and its returns. These factors contribute to reduce further students’ effort and hence their academic achievement, strengthening the stigmatization effect of lower tracking. Second, different levels of tracks are often associated with different levels of curricula ambition and availability of resources: lower tracks tend to lose on both fronts. Third, when tracking is the result of academic performance, both primary and secondary effects tend to strengthen educational inequalities between students with different socio-economic background. If policymakers opt for maintaining performance-bases tracking, it would be important to avoid early tracking, as social background tend to have a stronger effect at early ages.

When it comes to policies directed at widening access to (and increasing completion of) tertiary education, two main types of policy interventions^36^ can be distinguished. On the one hand we have “Outreach policies”, which are mainly of three types: (i) interventions that reduce information barriers of secondary education students on university curricula and costs, financial aid, and returns to higher education (including to specific fields of study); (ii) personalized assistance and guidance during the selection and application process; (iii) academic tutoring during upper-secondary education, with the purpose of increasing academic performance (and hence the chances of being admitted at a higher education institution). On the other hand, there are “Financial support” policies, which—in theory—would be especially beneficial for students from a low socio-economic background.

Herbaut and Geven (2020) provide an up-to-date review of the quasi-experimental literature on the impact that both types of policies have on the relationship between socio-economic background and the likelihood of enrolling and completing tertiary education. The main message from Herbaut and Geven’s review can be summarized as follows:

(a) information sessions on curricula, costs, financial aid, and returns to higher education work best when they are also accompanied by personalized assistance and guidance, as information alone –while it helps revising one’s beliefs—is often not sufficient to induce behavioral changes^37^ (this combined effect tends to be higher for disadvantaged students). In fact, a reduction of information asymmetries—in the absence of personalized assistance- often induces more efficient and realistic choices, but does not necessarily reduce the socio-economic gradient in higher education. These policies have been shown to work in relationship to enrollment (evidence on completion is minimal);

(b) financial support policies can have a sizeable impact on the reduction of the socio-economic gradient in higher education, but it is important to select the appropriate tool, and the appropriate size. Large universal grants have been shown to benefit the enrollment of (and completion by) disadvantaged students. The same applies to need-based grants/subsidies, as long as their amounts are able to cover a large part of the direct and indirect costs of higher education. The evidence on merit-based grants is generally pointing to negative effects on disadvantaged students’ enrollment, which is consistent with the fact that privileged students benefit from primary effects (i.e. they tend to have higher academic performance). Merit-based grants can work in reducing educational inequalities only if they are someway targeted to disadvantaged students (i.e. directed at high-performing students from a low socio-economic status). Tax incentives (e.g. tax credits for educational expenses) tend not to reduce the socio-economic gradient, while the scant evidence on loans points to a potential significant impact in reducing inequalities in enrollment.

Especially promising appear programs that combine financial aid with early commitments (i.e. the grant is a multiple of the amount of money saved by students during high-school to finance their tertiary education; see Azzolini et al., 2018). As for performance-based financial aid (i.e. financial support, conditional on achieving a given level of performance) and its impact on graduation rates of disadvantaged students, the quasi-experimental evidence is still scant but some studies show promising results^38^ (Binder et al., 2015; Erwin et al., 2021).

Finally, it important to improve participation to higher education by non-conventional students, adult learners, as well as by students with a vocational background^39^ (Katartzi & Hayward, 2020; Page & Scott-Clayton, 2016; Parry, 2016; Hoelsher et al., 2008).

The main objective of this study is to characterize inequality of opportunity in higher education in Europe in 2005 and 2011, and we do not attempt to derive robust policy implications, such as those summarized in the paragraphs above. However, we investigate the presence of significant statistical relationships between the estimated EIOp and a set of indicators that capture the country’s level of economic and human capital development, public expenditures on education (and specifically on tertiary education), variables proxying the characteristics and the quality of education and indicators of the returns from completing tertiary education. The evidence shows that real GDP per capita and EIOp are negatively correlated: one could read this as evidence supporting the hypothesis that higher equality of opportunity in tertiary education and GDP growth are complementary political objectives. We also find robust evidence for the variables capturing public expenditures in tertiary education and for one of the proxies for quality of higher education: higher spending on tertiary education (as a percentage of total spending or of GDP) has a strong negative association with EIOp, while a higher students/teacher ratio is strongly associated with higher EIOp.

Interventions aimed at reducing EIOp are desirable, as educational inequalities have an impact on future outcome achievements. Indeed, data show that there is a direct link between IOp in tertiary education and IOp in income. While guaranteeing a fair system for tertiary education is an important step towards the improvement of EOp in income, equality of opportunity should not be limited to the later stages of education, and in fact, should be guaranteed at every stage of the educational career, from early childhood education to upper secondary schooling. At the same time, it is important to remember that other factors may also affect the transmission of the beneficial effects of tertiary education attainment to the labour market, and hence co-determine labour market outcomes.

## Appendix

See Tables 7, 8, 9, 10, 11, 12, 13

### Annex 1. Probit Estimates for 2005

**Table 7:** Reduced-Form Regression of tertiary education achievement on Circumstances

Circumstances		AT	BE	CY	CZ	DE	DK
Gender	Male	0.239***	− 0.0373***	0.130***	0.114***	0.257***	− 0.262***
		(0.00157)	(0.00136)	(0.00525)	(0.00154)	(0.000442)	(0.00177)
Parental Education	M or F at least medium edu	0.336***	0.660***	0.803***	0.470***	0.236***	0.200***
		(0.00171)	(0.00164)	(0.00690)	(0.00271)	(0.000610)	(0.00203)
	F high edu	0.937***	1.288***	1.452***	1.254***	0.732***	0.787***
		(0.00398)	(0.00269)	(0.0137)	(0.00372)	(0.000723)	(0.00331)
	M high edu	1.419***	1.337***	1.386***	1.346***	0.598***	0.605***
		(0.00631)	(0.00385)	(0.0305)	(0.00553)	(0.00135)	(0.00401)
	Both high edu	1.403***	1.701***	1.603***	1.586***	0.825***	1.109***
		(0.0105)	(0.00345)	(0.0253)	(0.00490)	(0.00100)	(0.00419)
Parental Occupation	M or F at least bc	− 0.153***	− 0.0344***	− 0.222***	− 0.0667***	− 0.00491***	− 0.0507***
		(0.00263)	(0.00217)	(0.0109)	(0.00421)	(0.000777)	(0.00463)
	F wc	− 0.110***	0.0296***	− 0.160***	0.386***	0.0813***	0.0334***
		(0.00312)	(0.00283)	(0.0132)	(0.00255)	(0.000821)	(0.00459)
	M wc	0.0303***	0.140***	− 0.0776***	0.361***	0.303***	0.160***
		(0.00262)	(0.00212)	(0.0111)	(0.00328)	(0.000778)	(0.00463)
	Both white collar	0.0899***	0.238***	0.106***	0.788***	0.209***	0.275***
		(0.00288)	(0.00259)	(0.0128)	(0.00262)	(0.000845)	(0.00453)
Childhood financial hardship	Bad/Very Bad		− 0.307***	− 0.839***	− 0.154***		− 0.397***
			(0.00369)	(0.0157)	(0.00379)		(0.00506)
	Moderately bad		− 0.177***	− 0.803***	− 0.0404***		− 0.00402
			(0.00305)	(0.0123)	(0.00299)		(0.00423)
	Moderately good		− 0.214***	− 0.348***	0.0187***		0.0345***
			(0.00219)	(0.0105)	(0.00203)		(0.00256)
	Good		− 0.125***	− 0.250***	0.0365***		0.0322***
			(0.00222)	(0.0105)	(0.00205)		(0.00238)

Circumstances		EE	ES	FI	FR	GR	HU	IE
Gender	Male	− 0.501***	− 0.0249***	− 0.291***	− 0.155***	0.00103	− 0.124***	0.111***
		(0.00398)	(0.000625)	(0.00170)	(0.000575)	(0.00131)	(0.00150)	(0.00230)
Parental Education	M or F at least medium edu	0.401***	0.750***	0.276***	0.520***	0.701***	0.573***	0.876***
		(0.00488)	(0.00115)	(0.00215)	(0.000664)	(0.00202)	(0.00181)	(0.00310)
	F high edu	0.736***	1.104***	0.556***	1.230***	0.975***	1.327***	1.636***
		(0.00869)	(0.00127)	(0.00361)	(0.00138)	(0.00308)	(0.00326)	(0.00640)
	M high edu	0.833***	1.059***	0.639***	1.188***	1.051***	1.182***	0.838***
		(0.00722)	(0.00257)	(0.00398)	(0.00177)	(0.00567)	(0.00459)	(0.00677)
	Both high edu	1.336***	1.642***	0.704***	1.705***	1.618***	1.923***	1.620***
		(0.00871)	(0.00235)	(0.00401)	(0.00188)	(0.00533)	(0.00424)	(0.00815)
Parental Occupation	M or F at least bc	− 0.0961***	− 0.0154***	0.0192***	− 0.168***	− 0.183***	− 0.0996***	− 0.234***
		(0.00875)	(0.00114)	(0.00284)	(0.000968)	(0.00196)	(0.00269)	(0.00505)
	F wc	0.142***	0.00782***	0.259***	− 0.0562***	0.0421***	0.125***	− 0.0133**
		(0.00593)	(0.00147)	(0.00257)	(0.00105)	(0.00321)	(0.00226)	(0.00594)
	M wc	0.268***	0.132***	0.444***	0.135***	0.234***	0.0986***	-0.157***
		(0.00863)	(0.00114)	(0.00334)	(0.00103)	(0.00210)	(0.00285)	(0.00464)
	Both white collar	0.327***	0.0910***	0.390***	0.319***	0.259***	0.379***	0.132***
		(0.00702)	(0.00137)	(0.00308)	(0.00107)	(0.00299)	(0.00252)	(0.00540)
Childhood financial hardship	Bad/Very Bad	− 0.298***	− 0.519***	− 0.340***			− 0.209***	− 0.708***
		(0.0105)	(0.00120)	(0.00347)			(0.00272)	(0.00478)
	Moderately bad	− 0.212***	− 0.440***	− 0.173***			− 0.255***	− 0.497***
		(0.00676)	(0.00118)	(0.00296)			(0.00249)	(0.00432)
	Moderately good	− 0.105***	− 0.319***	− 0.00229			− 0.118***	− 0.277***
		(0.00526)	(0.000868)	(0.00223)			(0.00245)	(0.00307)
	Good	0.00855	− 0.141***	0.0527***			− 0.124***	− 0.210***
		(0.00579)	(0.000827)	(0.00234)			(0.00201)	(0.00303)

Circumstances		IS	IT	LT	LU	LV	NL	NO
Gender	Male	− 0.161***	− 0.0151***	− 0.376***	0.224***	− 0.616***	0.273***	− 0.122***
		(0.00805)	(0.000659)	(0.00240)	(0.00886)	(0.00354)	(0.00105)	(0.0460)
Parental Education	M or F at least medium edu	0.412***	0.754***	0.468***	0.437***	0.541***	0.440***	0.345***
		(0.00886)	(0.000841)	(0.00276)	(0.00960)	(0.00395)	(0.00137)	(0.0632)
	F high edu	1.039***	1.415***	0.993***	1.083***	1.073***	0.997***	0.763***
		(0.0160)	(0.00167)	(0.00588)	(0.0185)	(0.00807)	(0.00183)	(0.0966)
	M high edu	0.749***	1.083***	1.185***	0.855***	1.171***	0.954***	0.696***
		(0.0223)	(0.00303)	(0.00530)	(0.0362)	(0.00678)	(0.00335)	(0.0782)
	Both high edu	0.843***	1.820***	1.444***	3.484***	1.942***	1.405***	1.180***
		(0.0324)	(0.00308)	(0.00555)	(0.114)	(0.00861)	(0.00290)	(0.0948)
Parental Occupation	M or F at least bc	0.0984***	− 0.163***	0.173***	− 0.339***	− 0.209***	0.00412**	− 0.0881
		(0.0156)	(0.00104)	(0.00531)	(0.0207)	(0.00847)	(0.00195)	(0.0949)
	F wc	0.196***	− 0.000380	0.177***	− 0.0897***	0.0834***	0.154***	0.118
		(0.0149)	(0.00152)	(0.00399)	(0.0232)	(0.00562)	(0.00227)	(0.0897)
	M wc	0.221***	0.204***	0.399***	0.270***	0.222***	0.328***	0.390***
		(0.0154)	(0.00101)	(0.00508)	(0.0210)	(0.00872)	(0.00182)	(0.0976)
	Both white collar	0.509***	0.227***	0.370***	0.266***	0.198***	0.411***	0.429***
		(0.0150)	(0.00134)	(0.00423)	(0.0238)	(0.00640)	(0.00222)	(0.0927)
Childhood financial hardship	Bad/Very Bad	− 0.353***	− 0.595***	− 0.240***	− 0.131***	− 0.0586***	− 0.277***	− 0.135
		(0.0241)	(0.00131)	(0.00467)	(0.0255)	(0.00719)	(0.00276)	(0.139)
	Moderately bad	0.0377**	− 0.434***	− 0.273***	− 0.484***	0.0572***	− 0.201***	0.0179
		(0.0182)	(0.00112)	(0.00390)	(0.0199)	(0.00535)	(0.00204)	(0.109)
	Moderately good	− 0.186***	− 0.236***	0.0424***	− 0.156***	0.135***	0.0385***	0.0230
		(0.0120)	(0.000982)	(0.00330)	(0.0119)	(0.00456)	(0.00148)	(0.0702)
	Good	− 0.0425***	− 0.0816***	0.0899***	0.00744	0.110***	− 0.0675***	0.0329
		(0.0112)	(0.00102)	(0.00367)	(0.0120)	(0.00505)	(0.00143)	(0.0550)

Circumstances		PL	PT	SE	SI	SK	UK
Gender	Male	− 0.250***	− 0.331***	− 0.354***	− 0.238***	− 0.0358***	− 0.0633***
		(0.000772)	(0.00155)	(0.00191)	(0.00368)	(0.00192)	(0.000592)
Parental Education	M or F at least medium edu	0.635***	1.191***	0.591***	0.616***	0.443***	0.388***
		(0.000907)	(0.00353)	(0.00252)	(0.00447)	(0.00236)	(0.000769)
	F high edu	1.455***	1.027***	0.902***	0.854***	1.084***	0.901***
		(0.00199)	(0.00451)	(0.00357)	(0.00961)	(0.00395)	(0.00125)
	M high edu	1.492***	1.225***	0.649***	1.115***	1.271***	0.850***
		(0.00259)	(0.00515)	(0.00429)	(0.0160)	(0.00651)	(0.00141)
	Both high edu	2.047***	1.979***	1.242***	1.138***	1.529***	1.283***
		(0.00252)	(0.00616)	(0.00377)	(0.0140)	(0.00570)	(0.00168)
Parental Occupation	M or F at least bc	− 0.0422***	0.215***	− 0.194***	− 0.185***	− 0.235***	− 0.0853***
		(0.00125)	(0.00237)	(0.00376)	(0.00600)	(0.00421)	(0.00224)
	F wc	0.187***	0.191***	0.0129***	0.0300***	− 0.122***	− 0.0968***
		(0.00112)	(0.00275)	(0.00288)	(0.00593)	(0.00313)	(0.00175)
	M wc	0.125***	0.670***	0.0461***	0.0724***	− 0.0260***	0.0355***
		(0.00142)	(0.00236)	(0.00423)	(0.00657)	(0.00369)	(0.000752)
	Both white collar	0.385***	0.633***	0.312***	0.328***	0.175***	0.171***
		(0.00124)	(0.00256)	(0.00438)	(0.00605)	(0.00311)	(0.000775)
Childhood financial hardship	Bad/Very Bad	− 0.397***		− 0.357***	− 0.610***	0.193***	− 0.160***
		(0.00160)		(0.00536)	(0.00803)	(0.00557)	(0.00103)
	Moderately bad	− 0.308***		− 0.0985***	− 0.399***	0.123***	− 0.165***
		(0.00128)		(0.00392)	(0.00624)	(0.00549)	(0.00102)
	Moderately good	− 0.167***		− 0.212***	− 0.257***	0.0651***	− 0.139***
		(0.000990)		(0.00302)	(0.00533)	(0.00543)	(0.000804)
	Good	− 0.0751***		− 0.0580***	0.0272***	0.00649	− 0.0458***
		(0.00115)		(0.00247)	(0.00557)	(0.00573)	(0.000829)

Reference category for gender is “female”, for parental education is “both parents have low or no education”, for parental occupation is “both parents do not work or the information is unknows”, for “childhood financial problems” is “very good”. Standard errors in parentheses. Significance: ***p < 0.01, **p < 0.05, *p < 0.1

**Table 8:** Inequality of opportunity for tertiary education and contribution of each circumstance, 2005

Country	EIop	Gender	Parental education	Parental occupation	Childhood financial hardship
AT	17.08	16.73	56.18	27.09	n.a
BE	24.93	1.66	62.65	24.58	11.11
BG	n.a	n.a	n.a	n.a	n.a
CH	n.a	n.a	n.a	n.a	n.a
CY	26.71	1.40	53.79	21.79	23.02
CZ	32.24	3.65	34.30	51.07	10.97
DE	14.79	15.27	53.42	31.31	n.a
DK	17.90	14.94	48.90	30.73	5.43
EE	24.06	24.51	41.50	22.33	11.66
ES	20.89	1.44	53.03	19.46	26.07
FI	17.21	13.90	38.97	33.79	13.34
FR	24.68	5.81	61.07	33.12	n.a
GR	23.10	0.29	53.63	46.09	n.a
HR	n.a	n.a	n.a	n.a	n.a
HU	32.27	2.81	46.79	32.51	17.88
IE	26.30	5.33	58.52	12.19	23.96
IS	21.57	8.69	56.00	25.54	9.78
IT	35.37	0.64	47.63	27.82	23.90
LT	26.10	13.96	50.52	16.40	19.12
LU	30.78	6.13	38.29	43.20	12.38
LV	32.57	28.33	48.45	15.44	7.78
MT	n.a	n.a	n.a	n.a	n.a
NL	21.12	9.90	53.50	28.75	7.85
NO	19.12	3.06	48.12	42.14	6.67
PL	34.92	6.65	46.99	26.95	19.41
PT	32	12.28	37.43	50.29	n.a
SE	21.41	16.31	66.66	7.57	9.46
SI	36.48	7.86	41.36	26.14	24.64
SK	24.47	1.21	45.60	35.80	17.39
RO	n.a	n.a	n.a	n.a	n.a
UK	15.73	1.41	68.88	14.29	15.41

### Annex 2 Estimating EIOp Without Including “Financial Problem” as a Circumstance Variable

**Table 9:** Inequality of opportunity for tertiary education in 2005 and 2011

Country	EIOp 2005	EIOp 2011
AT	17.08	23.65
BE	24.86	22.50
BG	n.a	35.37
CH	n.a	18.40
CY	26.01	26.94
CZ	32.24	36.55
DE	14.79	20.40
DK	17.65	18.27
EE	23.87	21.89
ES	20.11	19.63
FI	16.51	15.30
FR	24.68	24.17
GR	23.10	25.10
HR	n.a	33.21
HU	31.50	36.15
IE	25.63	19.67
IS	18.93	18.71
IT	33.72	35.03
LT	25.40	26.17
LU	30.19	27.00
LV	32.49	25.49
MT	n.a	28.57
NL	20.84	20.64
NO	19.94	19.67
PL	34.26	31.56
PT	32.00	34.90
RO	n.a	38.23
SE	21.34	18.87
SI	34.84	22.93
SK	24.01	28.25
UK	15.67	16.68

### Annex 3 Estimating EIOp for Different Cohort Partitions

**Table 10:** Inequality of opportunity for tertiary education in 2011, analysis by cohort (25–35 vs. 35–60 and 25–50 vs. 50–60)

Country	EIOp Cohort 25–35	EIOp Cohort 35–60	EIOp Cohort 25–50	EIOp Cohort 50–60
AT	20.22	25.15	22.60	25.29
BE	19.48	24.02	20.65	26.91
BG	37.61	34.75	36.02	35.84
CH	16.25	20.01	17.17	22.21
CY	15.06	26.83	24.25	28.19
CZ	30.11	38.76	33.68	40.91
DE	19.74	21.02	19.51	22.90
DK	17.36	16.95	17.14	16.58
EE	21.35	22.82	23.25	19.37
ES	16.17	22.42	18.46	26.47
FI	16.15	16.13	14.23	18.08
FR	20.35	24.76	22.04	27.54
GR	23.29	23.49	24.59	24.60
HR	29.51	32.06	33.47	31.76
HU	34.11	36.35	35.18	36.52
IE	14.84	20.93	16.77	21.64
IS	22.88	18.82	18.16	21.63
IT	30.58	36.18	34.84	35.49
LT	19.46	25.74	24.94	24.99
LU	23.26	28.90	25.48	31.45
LV	23.01	26.39	24.42	28.73
MT	24.76	33.04	26.03	36.39
NL	19.64	20.90	20.09	21.57
NO	16.78	20.88	18.36	23.43
PL	22.05	35.18	26.93	36.93
PT	31.46	40.68	36.02	42.97
RO	39.20	33.01	40.14	27.80
SE	17.51	17.60	18.35	15.93
SI	17.69	26.06	21.71	25.30
SK	20.63	31.40	25.52	35.43
UK	13.87	18.20	15.89	19.52

**Table 11:** Inequality of opportunity for tertiary education in 2011, analysis by cohort (25–35 vs. 35–45 vs. 45–60 and 25–40 vs. 40–50 vs. 50–60)

Country	EIOp Cohort 25–35	EIOp Cohort 35–45	EIOp Cohort 45–60	EIOp Cohort 25–40	EIOp Cohort 40–50	EIOp Cohort 50–60
AT	20.22	26.02	24.12	21.57	25.00	25.29
BE	19.04	21.39	25.71	19.52	22.29	26.91
BG	37.61	34.00	36.33	36.11	35.88	35.84
CH	16.25	17.86	21.70	15.71	20.30	22.21
CY	15.06	25.03	25.87	16.73	28.53	28.19
CZ	30.11	37.18	40.13	31.81	36.80	40.91
DE	19.74	20.18	21.60	19.67	19.70	22.90
DK	17.36	15.04	17.20	15.85	18.42	16.58
EE	21.35	26.61	20.94	22.23	25.59	19.37
ES	16.17	17.85	26.06	16.92	20.35	26.47
FI	16.15	14.89	16.08	15.39	14.60	18.08
FR	20.35	20.23	27.78	19.31	23.78	27.54
GR	23.29	24.31	22.62	23.94	21.27	24.59
HR	29.51	34.39	33.67	29.27	39.49	31.76
HU	34.11	36.38	35.45	33.75	36.74	36.52
IE	14.84	15.91	22.90	12.80	21.90	21.64
IS	22.88	18.28	19.42	19.21	19.77	21.63
IT	30.58	36.77	35.44	32.20	36.83	35.49
LT	19.46	25.61	26.04	19.40	27.61	24.99
LU	23.26	26.57	31.84	24.89	26.54	31.45
LV	23.01	25.73	26.96	24.27	24.08	28.73
MT	24.76	28.68	34.94	24.95	32.77	36.39
NL	19.64	20.90	20.49	17.39	22.13	21.57
NO	16.77	21.13	21.65	15.73	21.46	23.43
PL	22.05	29.23	36.81	22.61	34.29	36.93
PT	31.46	39.48	42.08	33.54	39.41	42.97
RO	39.20	37.08	28.86	39.77	34.29	27.80
SE	17.51	18.76	15.17	17.04	17.59	15.92
SI	17.69	25.91	24.68	19.52	26.63	25.30
SK	20.63	31.07	33.92	22.88	31.18	35.43
UK	13.87	17.89	18.21	14.36	17.33	19.55

### Annex 4 Estimating EIOp Excluding Individuals Who Migrated Only as Adult

**Table 12:** Inequality of opportunity for tertiary education in 2011

Country	EIOp	Gender	Parental education	Parental occupation	Childhood financial hardship
AT	23.73	9.46	32.66	43.60	14.29
BE	22.48	5.19	53.13	29.55	12.12
BG	35.71	15.84	43.40	31.16	9.60
CH	18.32	29.78	32.58	31.33	6.31
CY	27.69	2.56	46.44	31.37	19.62
CZ	36.50	0.15	56.36	33.98	9.50
DE	20.62	11.68	55.50	22.83	9.99
DK	18.97	8.49	45.76	34.21	11.54
EE	22.08	30.47	35.77	25.91	7.84
ES	21.02	4.38	35.01	38.70	21.90
FI	15.12	30.48	47.70	10.05	11.77
FR	24.57	4.30	50.01	33.57	12.12
GR	24.94	1.11	52.74	30.44	15.71
HR	33.39	7.92	45.12	32.26	14.71
HU	36.31	8.98	45.41	33.46	12.15
IE	20.06	3.70	45.02	37.05	14.24
IS	20.93	29.89	37.60	20.80	11.71
IT	35.41	3.50	45.83	34.30	16.37
LT	25.62	8.68	49.09	27.64	14.59
LU	27.52	3.54	33.45	44.18	18.83
LV	25.73	28.01	32.82	30.11	9.06
MT	29.73	0.69	47.80	32.46	19.05
NL	20.60	4.96	50.11	38.86	6.06
NO	18.66	11.74	53.99	32.90	1.37
PL	32.25	7.68	38.83	38.66	14.83
PT	37.18	13.86	20.99	33.98	31.17
RO	38.44	1.59	52.80	29.57	16.04
SE	17.72	25.60	55.68	14.68	4.04
SI	25.75	12.01	41.41	34.52	12.05
SK	28.54	4.41	35.29	45.88	14.42
UK	16.96	0.81	52.42	34.74	11.99

### Annex 5 Estimating EIOp Including Second Generation Migrant as Additional Circumstances

**Table 13:** Inequality of opportunity for tertiary education in 2011

Country	EIOp	Gender	Parental education	Parental occupation	Second generation migration status	Childhood financial hardship
AT	23.75	9.39	32.60	43.50	0.26	14.24
BE	22.49	4.80	51.42	27.76	5.13	10.89
BG	35.72	15.54	43.13	30.90	1.12	9.31
CH	18.23	29.53	32.29	31.12	0.83	6.23
CY	27.60	2.40	46.40	30.98	0.60	19.62
CZ	36.72	0.32	55.74	33.03	2.08	8.83
DE	20.50	11.21	54.85	21.94	1.98	10.03
DK	18.71	8.39	45.11	33.61	1.04	11.85
EE	22.21	28.85	34.03	24.69	5.23	7.19
ES	20.86	4.73	35.13	38.22	0.05	21.87
FI	15.32	27.81	46.55	9.00	6.23	10.42
FR	24.51	4.58	49.83	33.48	0.15	11.97
GR	25.27	1.11	52.14	30.53	0.51	15.71
HR	33.43	7.56	44.61	31.78	1.73	14.32
HU	36.32	8.95	45.38	33.43	0.12	12.12
IE	20.05	3.70	44.53	36.63	0.86	14.26
IS	19.54	31.57	34.06	21.04	2.18	11.15
IT	35.42	3.36	45.61	34.07	0.81	16.15
LT	25.67	8.34	48.73	27.33	1.37	14.23
LU	27.46	3.02	32.47	43.44	3.07	17.99
LV	26.08	25.58	31.69	28.10	7.02	7.61
MT	29.77	0.29	46.46	32.18	0.87	20.19
NL	20.65	4.82	50.01	39.06	0.30	5.81
NO	18.78	8.47	46.95	36.22	0.41	7.94
PL	32.24	7.57	38.71	38.57	0.45	14.69
PT	37.82	13.92	20.72	34.04	0.67	30.66
RO	38.43	1.34	52.13	29.32	1.70	15.50
SE	18.96	25.27	54.39	14.04	2.47	3.83
SI	23.32	14.09	40.04	33.49	1.11	11.27
SK	28.47	4.25	34.99	45.68	0.75	14.34
UK	17.08	0.72	50.33	33.30	4.23	11.42

## Footnotes

1 On the role of education for social mobility see Stuhler (2018).

2 See the seminal contributions by Fleurbay (1994), Roemer (1993, 1998), and Van de Gaer (1993). See also Ferreira and Peragine (2016), Ramos and Van de gaer (2016) and Roemer and Trannoy (2015) for recent surveys of the literature.

3 As a consequence, completion of primary and (lower) secondary education is less and less used as a predictor of future earnings. In addition, it is often difficult to estimate returns to primary and (lower) secondary education since the comparison group, that is, workers with no education, is almost empty.

4 The estimations presented in Psacharopoulos and Patrinos (2018) will be part of the World Bank forthcoming dataset on educational statistics (EdStats).

5 For a discussion of this important point see Roemer (1998) and Brunori et al. (2012).

6 For a review of the sociological approach to inequality of educational opportunity see Erikson (2019).

7 Heisig et al. (2020) compare the size of the socioeconomic gradient in educational attainment to that in achievement (i.e. competencies), using PIAAC data from the first and second round for 23 countries. The evidence shows that the association with social background is stronger for attainment than for achievement in all the countries.

8 For a review see Farkas (2018).

9 These metrics assess both cognitive (i.e. math reading, and science) and non-cognitive skills (such as conscientiousness and persistence). This might explain why non-cognitive skills are found to account for an important share of across country variation in standardized tests (e.g. PISA) scores (see Zamarro et al., 2019). For a review of the role of non-cognitive skills over the life-cycle see Lechner et al. (2019).

10 Well-off parents are often part of social networks that provide opportunities for access to highly paid occupations, which rises the incentive for their offsprings to acquire the necessary levels of education (compared to students from a disadvantaged background: for a recent discussion of the importance of social networks on inequality see Jackson, 2021). This effect tends to rise in labour markets characterized by high barriers to entry and high importance of personal contacts.

11 Boliver (2011) shows that such qualitative elements have been fundamental in maintaining and strengthening inequality of opportunity in British higher education, during the period 1960–1995. Similar evidence is found by Galindo-Rueda et al. (2004) and Machin and Vignoles (2004). For more recent evidence broadly confirming these results for the U.K. see Crawford et al. (2016). See also Vignoles and Murray (2016).

12 This has the implication that researchers should focus their attention on transitions between different types/levels of education. For a discussion of education as a lifelong process see Blossfeld et al. (2019).

13 See Bukodi and Goldthorpe (2013) for a discussion of how the choice of the variables for the measurement of social origin can affect the results.

14 Shavit and Blossfeld (1993) is the first attempt to analyse inequality of educational opportunity in a comparative framework. The main conclusion from this book is that the association between social origin and educational attainment has remained fairly stable after World War II in 11 over the 13 industrialized countries analysed (the exceptions are Sweden and the Netherlands). However, such findings have been challenged on the methodological ground by Breen and co-authors (see Breen and Jonsson, 2005; Breen et al., 2009).

15 See Bar-Haim and Shavit (2013) and Van de Werfhorst and Mijs (2010). For a policy-oriented review of recent contributions see Caturianas et al. (2017).

16 The dependent variable is not overall educational inequality but only the part that arises from inequality of opportunity (EIOp).

17 The existing literature has developed two main approaches to the measurement of opportunity inequality, namely the ex-ante and the ex-post approach. According to the ex-ante approach, there is equality of opportunity if the set of opportunities is the same for all individuals, regardless of their circumstances. Hence, in the ex-ante version, the compensation principle is formulated with respect to individual opportunity sets: it requires reducing the inequality between opportunity sets. On the other side, according to the ex-post approach, there is equality of opportunity if and only if all those who exert the same effort end up with the same outcome. The compensation principle, in the ex-post version, is thus defined with respect to individuals with the same effort but different outcomes: it requires reducing outcome inequality among the individuals with the same effort. See Ferreira and Peragine (2016) and Roemer and Trannoy (2015).

18 For instance, suppose we have three circumstances—parental education, parental occupation and birthplace—and we want to assess the contribution of parental occupation to EIOp. We should make the following three comparisons: compare EIOp obtained on the base of a partition into types that are constructed using all the three circumstances to EIOp obtained on the base of a partition into types constructed using parental education and birthplace only; compare EIOp using parental education and parental occupation to EIOp obtained using parental education; compare EIOp estimated using birthplace and parental occupation and EIOp computed using birthplace; compare EIOp using parental occupation and EIOp using no circumstances. The average across all four comparisons represents the marginal contribution of parental occupation to EIOp (for details on the methodology see Brunori et al., 2019).

19 The 2005 wave consists of the following countries: Austria (AT), Belgium (BE), Cyprus (CY), Czech Republic (CZ), Germany (DE), Denmark (DK), Estonia (EE), Greece (EL), Spain (ES), Finland (FI), France (FR), Hungary (HU), Ireland (IE), Iceland (IS), Italy (IT), Lithuania (LT), Luxemburg (LU), Latvia (LV), Netherlands (NL), Norway (NO), Poland (PL), Portugal (PT), Sweden (SE), Slovenia (SI), Slovakia (SK) and Great Britain (UK). In the 2011, Bulgaria (BG), Switzerland (CH), Croatia (HR), Malta (MT) and Romania (RO) are added to the previous list, counting 31 countries in total.

20 The age in which migrants reach the destination country is available only for 2011. This allows us to include (non-adult) migrants, but only for 2011, in the robustness analysis.

21 We are aware that socio-economic background is also a determinant of the field of study and institution in which an individual will get her/his tertiary education degree. Unfortunately, these kinds of information are not available in EU-SILC database. Therefore, in this study we consider only the impact of different circumstances on the probability of completing tertiary education.

22 Information about mother’s and father’s education is registered in EU-SILC on the base of the ISCED-97 code. "Low level" of education corresponds to levels 0, 1, and 2 of ISCED-97. "Medium level" and "High level" of education correspond, respectively, to levels 3 and 4, and to levels 5 and 6 of ISCED-97.

23 Information about mother’s and father’s occupation is registered in EU-SILC on the base of the ISCO-88 code in 2011 and ISCO-08 code in 2005. In particular, blue collar encompasses the following codes: Elementary occupation, Plant Operator, Craft/Trades occupation, Agriculture occupation. White collar encompasses: occupation in service, Clerical work, Technician, Professional, Manager. The remaining is composed by individuals for which information on mother or father occupation is missing, or their mother or father is dead or not working or unknown.

24 Notice that information about this circumstance is missing in the 2005 wave for Austria, Germany, France, Greece and Portugal.

25 http://data.uis.unesco.org/.

26 In fact, women are much less represented than men in STEM. Using graduation data from UNESCO, Stoet and Geary (2018) find that, between 2012 and 2015, the propensity of women relative to men to graduate with STEM degrees ranged from 12.4 in Macao to 40.7 in Algeria; the median propensity was 25.4. For instance, it is about 25% in France, 30% in UK and 35% in Italy.

27 To make the comparisons more readable we consider jointly the contributions from parental education and parental occupation.

28 Our results are not directly comparable with those of Triventi (2013) since we use a different variable to capture inequality of opportunity, as well as a different variable to represent the “competition hypothesis”. We also have a larger number of observations (roughly four times higher). On the theoretical level, and to be fair with the “competition hypothesis”, we should take into account that not only the demand for tertiary education has increased, but also its supply. Social stratification could hence lead to a “quality segmentation”, with students from affluent parents graduating from more prestigious universities, without necessarily affecting the “quantitative dimension” (i.e. social gap in the likelihood of graduating). However, with EU-SILC data we cannot identify the “quality” of the higher education institutions from which students graduate.

29 A symmetric – and not significant—result is found for the share of all secondary education students enrolled in general programmes.

30 E.g.: government expenditures in education and government expenditures in tertiary education (*r* = 0.89); relative unemployment rate and relative employment rate (*r* = 0.42); real per capita GDP and government expenditures in tertiary education (*r* = 0.54).

31 Analogous results hold when we use public expenditure on tertiary education as a ratio of total public expenditure.

32 The relative unemployment rate is the variable that allows us to use the largest number of observations in the estimation.

33 Suhonen and Karhunen (2019) estimate a positive causal effects of parental education on their offsprings’ education.

34 The distinction between primary and secondary effects becomes blurry when one realizes that effort, which also depends upon educational expectations (i.e. a secondary effect), affects academic achievement (i.e. performance).

35 Covid-19 is likely to have exacerbated pre-existing inequalities: see Di Pietro et al. (2020).

36 In both cases these are policies that address mostly the secondary effects of the socio-economic background.

37 See also Barone et al. (2017), Barone et al. (2018), and Abbiati et al. (2018).

38 Vignoles and Powdthavee (2009) points to the importance of policies supporting completions of higher education by disadvantaged students.

39 For a review and discussion of policies for inclusion in higher education in the EU see Kottmann et al. (2019), and Orr et al., (2017a, 2017b).
